# Decrease of *SYNGAP1* in GABAergic cells impairs inhibitory synapse connectivity, synaptic inhibition and cognitive function

**DOI:** 10.1038/ncomms13340

**Published:** 2016-11-09

**Authors:** Martin H. Berryer, Bidisha Chattopadhyaya, Paul Xing, Ilse Riebe, Ciprian Bosoi, Nathalie Sanon, Judith Antoine-Bertrand, Maxime Lévesque, Massimo Avoli, Fadi F. Hamdan, Lionel Carmant, Nathalie Lamarche-Vane, Jean-Claude Lacaille, Jacques L. Michaud, Graziella Di Cristo

**Affiliations:** 1 Department of Neurosciences, Université de Montréal, C.P. 6128, Succ. Centre-Ville, Montréal, Quebec, Canada H3C 3J7; 2 CHU Sainte-Justine Research Center, 3175 Côte-ste-Catherine, Montréal, Quebec, Canada H3T 1C5; 3 Groupe de Recherche sur le Système Nerveux Central, Université de Montréal, C.P. 6128, succ. Centre-ville, Montréal, Quebec, Canada H3C 3J7; 4 Department of Anatomy and Cell Biology, Cancer Research Program of the Research Institute of MUHC, McGill University, 2155 Guy Street, Suite 500, Montreal, Quebec, Canada H3H 2R9; 5 Department of Neurology and Neurosurgery, Montreal Neurological Institute, McGill University, 3801 University Street, Montreal, Quebec, Canada H3A 2B4; 6 Department of Pediatrics, Université de Montréal, CHU Sainte-Justine, 3175, chemin de la Côte-Sainte-Catherine, 7th floor, block 9, Montreal, Quebec, Canada H3T 1C5

## Abstract

Haploinsufficiency of the *SYNGAP1* gene, which codes for a Ras GTPase-activating protein, impairs cognition both in humans and in mice. Decrease of *Syngap1* in mice has been previously shown to cause cognitive deficits at least in part by inducing alterations in glutamatergic neurotransmission and premature maturation of excitatory connections. Whether *Syngap1* plays a role in the development of cortical GABAergic connectivity and function remains unclear. Here, we show that *Syngap1* haploinsufficiency significantly reduces the formation of perisomatic innervations by parvalbumin-positive basket cells, a major population of GABAergic neurons, in a cell-autonomous manner. We further show that *Syngap1* haploinsufficiency in GABAergic cells derived from the medial ganglionic eminence impairs their connectivity, reduces inhibitory synaptic activity and cortical gamma oscillation power, and causes cognitive deficits. Our results indicate that *Syngap1* plays a critical role in GABAergic circuit function and further suggest that *Syngap1* haploinsufficiency in GABAergic circuits may contribute to cognitive deficits.

## Abstract (web summary)

Glutamatergic signalling regulation by Syngap1 has been linked to intellectual disabilities. Here, the authors find Syngap1 also regulates cortical GABAergic synaptic signalling development and that this reduced inhibitory signalling contributes to cognitive deficits in a mouse model.

## Graphical abstract

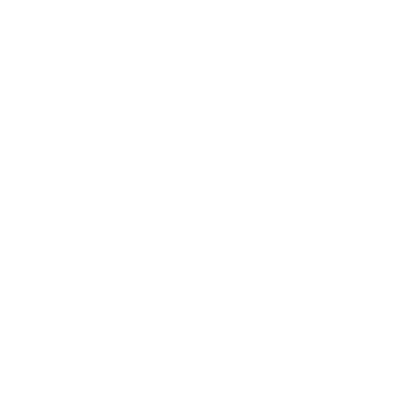

Long-term changes in the strength of synaptic transmission are thought to be critical both during brain development and for learning and memory throughout life. The Ras family GTPases, their downstream signalling proteins and upstream regulators are key biochemical cascades modulating synaptic plasticity. *SYNGAP1* codes for a GTPase-activating protein (GAP) that physically interacts with the small GTPase Ras, which in turn acts in a cycle as a molecular switch with an active GTP-bound form and an inactive GDP-bound form^1^,^2^. Ras has a slow intrinsic GTPase activity, and GAPs such as SYNGAP1 negatively regulate Ras by enhancing the hydrolysis of GTP to GDP. The importance of SYNGAP1 in synaptic plasticity is exemplified by the fact that *de novo* mutations in the *SYNGAP1* gene cause moderate or severe intellectual deficiency (ID)^3^,^4^,^5^,^6^,^7^,^8^,^9^. SYNGAP1 function has been mainly studied in excitatory neurons. For example, in primary neuronal cultures, SYNGAP1 functions to limit excitatory synapse strength by restricting the expression of the AMPA receptor (AMPAR) at the postsynaptic membrane^1^,^2^,^10^,^11^. In mice, *Syngap1* haploinsufficiency causes abnormal synaptic plasticity as well as behavioural abnormalities and cognitive deficits^12^,^13^,^14^,^15^. *Syngap1*^*+/−*^ mice are also characterized by enhanced excitatory synaptic transmission early in life and the premature maturation of glutamatergic synapses^16^,^17^. Thus, it has been proposed that glutamatergic synaptic alterations represent the main contributing factor for the occurrence of cognitive and behavioural deficits^16^,^17^.

During healthy cortical network activity, excitation is precisely balanced by GABAergic inhibition. Inhibitory activity not only regulates circuit excitability, but also restricts the temporal window for integration of excitatory synaptic inputs and resulting spike generation, thereby facilitating an accurate encoding of information in the brain^18^. In addition, GABAergic cells are implicated in generating temporal synchrony and oscillations among networks of pyramidal neurons, which are involved in complex cognitive functions, such as perception and memory^19^,^20^. Furthermore, GABAergic inhibition plays a critical role in modulating developmental plasticity in the young brain^21^. Highlighting the importance of GABA interneurons in cognitive functions, cortical circuits in several mouse models of ID and autistic-like behaviour show excitation/inhibition imbalance, which is due to alterations in glutamatergic or GABAergic neurotransmission, or more often, in both^16^,^22^,^23^,^24^,^25^,^26^,^27^. Whether and to what extent *Syngap1* haploinsufficiency affects GABAergic cell circuits, thus contributing to excitation/inhibition imbalance and cognitive abnormalities remains unclear.

Here, we examined the specific contribution of *Syngap1* to the formation of perisomatic innervations by parvalbumin-positive basket cells, a major population of GABAergic neurons, by single-cell deletion of *Syngap1* in cortical organotypic cultures. In addition, we generated mice with specific deletion of *Syngap1* in GABAergic neurons generated in the medial ganglionic eminence (MGE) to assess its role in the establishment of mature GABAergic connectivity and mouse cognitive function *in vivo.* We found that *Syngap1* strongly modulated the formation of GABAergic synaptic connectivity and function and that MGE cell-type specific *Syngap1* haploinsufficiency altered cognition.

## Results

### Single-cell Syngap1 knockdown reduced PV+ cell innervations

*Syngap1* expression peaks when the processes of synaptogenesis and developmental plasticity are heightened^28^. While its expression in glutamatergic cell is well documented^1^,^14^,^15^,^16^,^29^,^30^,^31^,^32^, few studies have also reported SYNGAP1 expression in GABAergic neurons^17^,^33^,^34^. To confirm that SYNGAP1 is present in GABAergic neurons, we prepared dissociated neuronal cultures from E18 wild-type embryos and immunostained them for GAD67, which is the main GABA synthesizing enzyme^35^, and SYNGAP1 at DIV21, after the peak of synapse formation. We found that GAD67-positive cells co-localized with SYNGAP1 (Supplementary Fig. 1a, 63±5% co-localization), indicating that SYNGAP1 is indeed expressed by GABAergic neurons.

GABAergic circuits comprise an astonishing variety of different cell types, exhibiting differences in molecular, morphological and electrophysiological properties^19^. These differences are particularly important in the light of recent discoveries suggesting that different GABAergic cell types are recruited by different behavioural events^19^. Among the different GABAergic neuron subtypes, the parvalbumin-expressing (PV+) basket cells comprise the largest subpopulation in cortical circuits^19^. Each PV+ basket cell innervates hundreds of neurons, with large, clustered boutons targeting the soma and the proximal dendrites of postsynaptic targets, an optimal location to control timing and frequency of action potential generation^19^,^36^. Such distinct features of PV+ basket cell innervations are achieved during the first postnatal month in rodents and are modulated by neural activity levels^35^,^37^,^38^,^39^. We found that almost the totality of PV+ basket cells express SYNGAP1 in dissociated neuronal cultures (Supplementary Fig. 1b) and thus we sought to investigate whether *Syngap1* plays a role in the formation of the innervation of PV+ basket cells, by inducing single-cell *Syngap1* deletion in cortical organotypic cultures.

To reduce *Syngap1* expression in isolated PV+ basket cells and simultaneously label their axonal arbours at synaptic resolution, we used a previously characterized promoter region P_G67_ (ref. 37) to express either the Cre recombinase together with GFP (P_G67_-GFP/Cre) or GFP alone (P_G67_-GFP; control basket cells) in cortical organotypic cultures prepared from *Syngap1*^*flox/flox*^ mice^35^,^37^,^38^,^39^,^40^. In organotypic cultures, PV+ basket cells initially display very sparse and simple axons, which develop into complex and highly branched arbours in the following 4 weeks, recapitulating the *in vivo* situation^37^,^38^. We chose to induce *Syngap1* deletion in PV+ basket cells at Equivalent Postnatal day 10 (EP10=cultured prepared at Postnatal day 4+6DIV) and collect the cultures at EP24 because extensive and stereotyped maturation of PV+ basket cell innervations occurs during this time window^37^,^37^,^38^,^39^,^40^,^41^.

We investigated two aspects of PV+ basket cell axon innervation: (1) the extent of perisomatic innervation around single neuronal somata (terminal branching and perisomatic bouton density) and (2) the fraction of potentially innervated cells in the field (percentage of innervation). By studying the localization of pre- and post-synaptic markers and performing electron microscopy, we have previously shown that the vast majority of GFP-labelled boutons in our experimental condition represent presynaptic terminals^35^,^37^,^42^.

**Figure 1:**
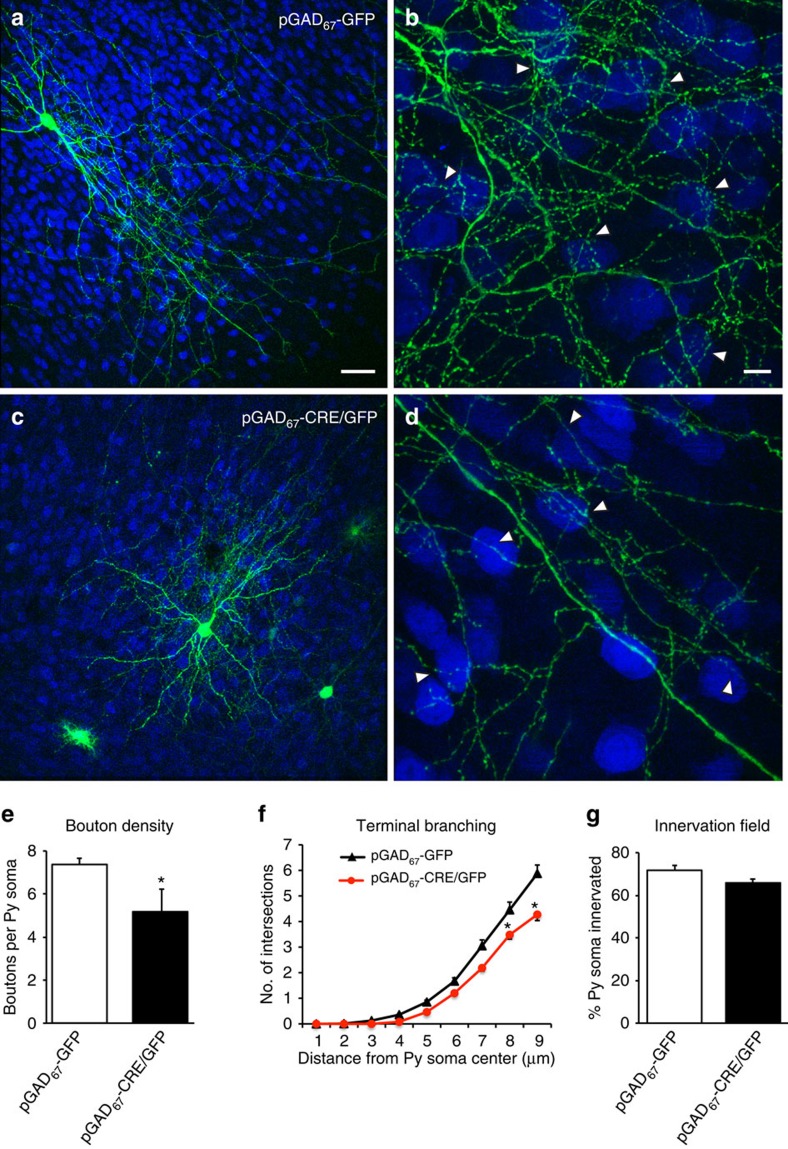
Single-cell Cre-mediated inactivation of *Syngap1* reduces the number of cortical GABAergic basket cell boutons and terminal branching. (**a**) Control GABAergic basket cell transfected with pGAD_67_-GFP (green) in EP24 cortical organotypic cultures from *Syngap1*^*flox/flox*^ mice showing (**b**) rich axonal branching and numerous clustered boutons (arrowheads) around NeuN-positive somata (blue). (**c**,**d**) Conversely, a basket cell transfected with pGAD_67_-Cre/GFP from EP16-24 shows markedly reduced axonal branching and perisomatic boutons (arrowheads). (**e**) Bouton density and (**f**) complexity of terminal axon branching are significantly decreased in pGAD_67_-Cre/GFP basket cells (**e**: Student's *t*-test, *P=0.002; **f**: Student's *t*-test, *P*=0.045 at 8 μm and *P*=0.010 at 9 μm), whereas the percentage of innervated cells (**g**) is not affected compared with age-matched control basket cells (Student's *t*-test, *P*=0.125). *n*=8 basket cells transfected with pGAD_67_-GFP from *n*=5 mice; *n*=6 basket cells transfected with pGAD_67_-Cre/GFP from *n*=5 mice. Scale bar, (**a**,**c**) 50 μm; (**b**,**d**) 10 μm.

Whereas control basket cells at EP24 showed complex perisomatic innervations (Fig. 1a,b), *Syngap1* knockdown in single-basket cells from EP10-24 induced a significant reduction in the number of both axonal branching and synaptic boutons innervating the target neurons (NeuN-positive cells; Fig. 1c–f: boutons/soma, 7.4±0.3 for control versus 5.2±0.5 for basket cells transfected with P_G67_-GFP/Cre; Students *t*-test *P*=0.002). On the other hand, the percentage of potentially innervated neurons were not significantly different between the two groups (Fig. 1g: 71%±2 for control versus 66%±2 for basket cells transfected with P_G67_-GFP/Cre; Students *t*-test, *P*=0.125), suggesting that *Syngap1* deletion specifically affected local synapse formation of PV+ basket cells but not their overall axonal growth. *Syngap1* knockout at later age (EP16-24) caused a similar decrease in perisomatic bouton density (Supplementary Fig. 2, boutons/soma, 7.4±0.3 for control versus 4.7±0.5 for basket cells transfected with P_G67_-GFP/Cre; Students *t*-test *P*=0.00023). Altogether, these data demonstrate that *Syngap1* promotes the formation of PV+ basket cell innervations in a cell-autonomous manner.

### MGE-specific Syngap1 knockdown impaired PV cell connectivity

Our data suggest that reducing *Syngap1* expression has a direct impact on the formation of PV+ basket cell innervations *in vitro.* To investigate the role of *Syngap1* in PV+ cell circuits *in vivo*, we generated mice that were heterozygous or homozygous for the *Syngap1*^*flox*^ allele and hemizygous for the Tg(*Nkx2.1-Cre*) transgene. This approach allowed the conditional deletion of *Syngap1* in cortical,^43^ hypothalamic^43^ and mesencephalic^44^ (striatum) GABAergic interneurons originating from the MGE as early as embryonic day 10.5 and throughout adulthood^45^. In mouse, Nkx2.1-expressing MGE precursors produce most of PV+ and Somatostatin (SST)+ cortical interneurons^45^. To confirm the specificity of CRE expression, we fluorescently labelled the cells expressing Cre by crossing Tg(*Nkx2.1-Cre*);*Syngap1*^*flox/+*^ mice with a GFP reporter line (RCE mouse^46^). We confirmed that cortical GFP+ cells expressed either PV or SST (Supplementary Fig. 3; percentage of GFP+ cells expressing PV or SST, 74±7% and 25±6%, respectively, *n*=3 mice). Further, the large majority of cortical neurons immunopositive for PV also expressed GFP (85±6%, *n*=3 mice), as previously reported^47^.

We focused our study on Tg(*Nkx2.1-Cre*);*Syngap1*^*flox/+*^mice, since most of Tg(*Nkx2.1-Cre*);*Syngap1*^*flox/flox*^ mice died at birth (9 out of 11, Tg(*Nkx2.1-Cre*);*Syngap1*^*flox/flox*^ versus none of Tg(*Nkx2.1-Cre*);*Syngap1*^*flox/+*^ mice) as it is the case for *Syngap1*^*−/−*^ mice. Therefore, *Syngap1* expression in MGE-derived cells may be required for survival. Alternatively, since both *Syngap1* and *Nkx2.1* are expressed in the developing lungs and heart^28^,^29^,^45^, it is possible that the deletion of *Syngap1* outside the brain is the cause underlying this early mortality.

**Figure 2:**
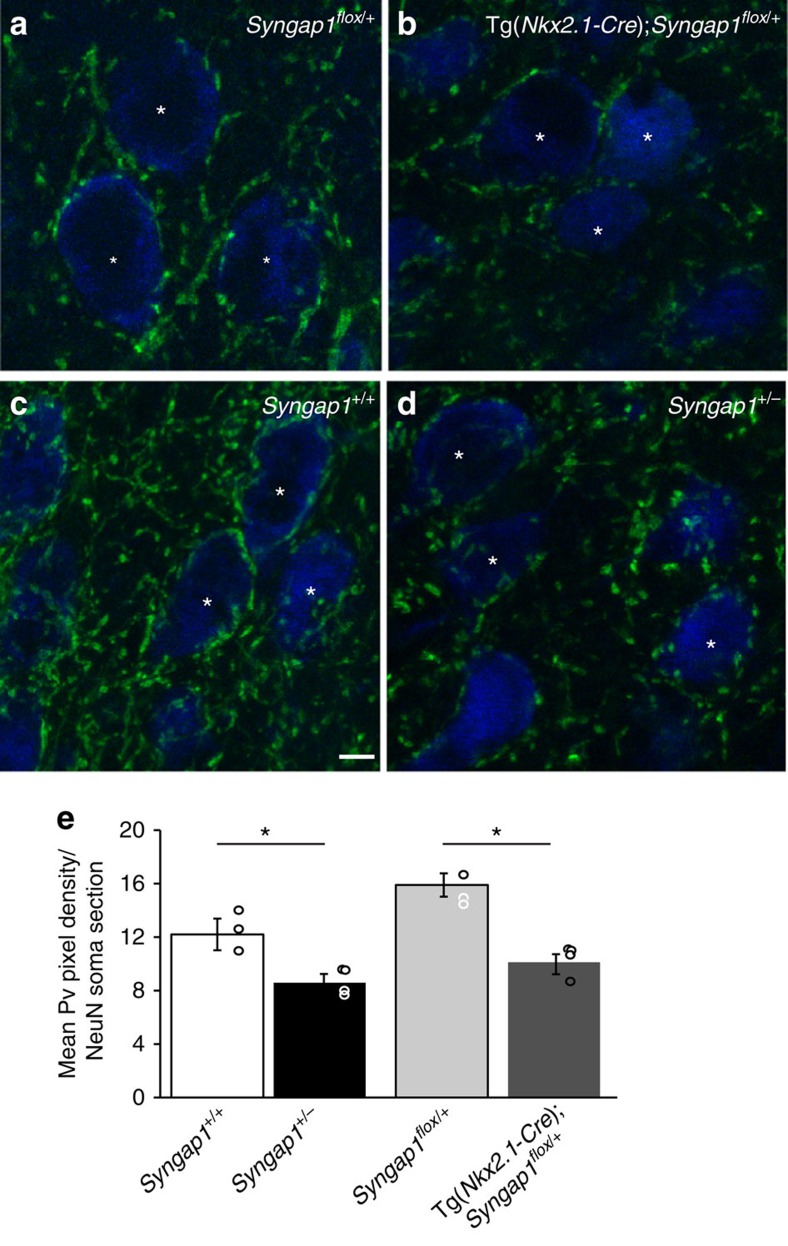
Both MGE-specific and germ-line *Syngap1* haploinsufficiency reduce PV+ perisomatic innervation in the cortex of young adult mice. (**a**,**b**) Confocal images of layer 5 pyramidal cells in somatosensory cortex of P45 mice show distinct and intense PV-positive perisomatic rings (green), which enclosed a large portion of neuronal somata (NeuN, blue) in control *Syngap1*^*flox/+*^ mice (**a**), whereas in Tg(*Nkx2.1-Cre*);*Syngap1*^*flox/+*^ mice (**b**) perisomatic PV-ring are less intense and often do not form a complete ring around cell somata. (**c**,**d**) Similar differences can be observed between *Syngap1*^*+/+*^(**c**) and *Syngap1*^*+/−*^ (**d**) mice. (**e**) Quantification show that mean PV fluorescence per NeuN soma section is significantly reduced in Tg(*Nkx2.1-Cre*);*Syngap1*^*flox/+*^ and *Syngap1*^*+/−*^ compared with their respective control littermates (One-way ANOVA with Dunn's multiple comparisons *post hoc* method, *P=0.00404 for Tg(*Nkx2.1-Cre*);*Syngap1*^*flox/+*^ versus *Syngap1*^*flox/+*^, *P=0.0274 versus *Syngap1*^*+/−*^ for *Syngap1*^*+/+*^). Graph bars represent mean±s.e.m., circles represent values for each analyzed mouse. *n*=91 neurons from 3 *Syngap1*^*flox/+*^ mice; *n*=93 neurons from 4 Tg(*Nkx2.1-Cre*);*Syngap1*^*flox/+*^ mice, *n*=100 neurons from 4 *Syngap1*^*+/+*^, *n*=109 neurons from 4 *Syngap1*^*+/−*^. Scale bar, 10 μm.

First, we investigated whether *Syngap1* haploinsufficiency restricted to MGE-derived GABAergic cells affected perisomatic innervations formed by PV+ basket cells in the cortex. In particular, we analyzed the intensity of PV immunostaining around layer 5 NeuN-positive neurons in the somatosensory cortex of P45 Tg(*Nkx2.1-Cre*);*Syngap1*^*flox/+*^ and control littermates as reported in^27^. In control mice, distinct PV-positive perisomatic bouton rings enclosed a large portion of the pyramidal cell soma, while PV-positive perisomatic rings were significantly less developed in Tg(*Nkx2.1-Cre*);*Syngap1*^*flox/+*^(Fig. 2a,b,e: PV mean intensity (a.u.), 15.9±0.9 for *Syngap1*^*flox/+*^ mice versus 10.1±0.8 for Tg(*Nkx2.1-Cre*);*Syngap1*^*flox/+*^mice; One-way ANOVA *post hoc* Dunn's test, *P*=0.00404), which suggests a decrease of perisomatic boutons made by PV+ basket cells. This effect was not restricted to the cortex, as we found a similar reduction in PV staining intensity around hippocampal CA1 pyramidal cells (PV mean intensity (a.u.), 17±2, *n*=52 cells from three *Syngap1*^*flox/+*^ mice versus 9.9±0.9, *n*=56 cells from four Tg(*Nkx2.1-Cre*);*Syngap1*^*flox/+*^mice; one-way ANOVA, *post hoc* Dunn's test, *P*=0.0183).

To evaluate whether germ-line *Syngap1* haploinsufficiency affects GABAergic perisomatic innervation formed by PV+ basket cells in the cortex, we performed the same analysis in *Syngap1*^*+/−*^ and control littermates. We found a reduction in perisomatic PV-positive rings in the somatosensory cortex of *Syngap1*^*+/−*^ mice, which was comparable to what we observed in Tg(*Nkx2.1-Cre*);*Syngap1*^*flox/+*^ (Fig. 2c–e; PV mean intensity (a.u.), 12±1, versus 8.6±0.7 from *Syngap1*^*+/+*^ and *Syngap1*^*+/−*^mice, respectively; one-way ANOVA, *post hoc* Dunn's test *P*=0.0274). Thus, *Syngap1* haploinsufficiency both in germ line and MGE-derived interneurons reduced cortical PV+ basket cell bouton density *in vivo.*

**Figure 3:**
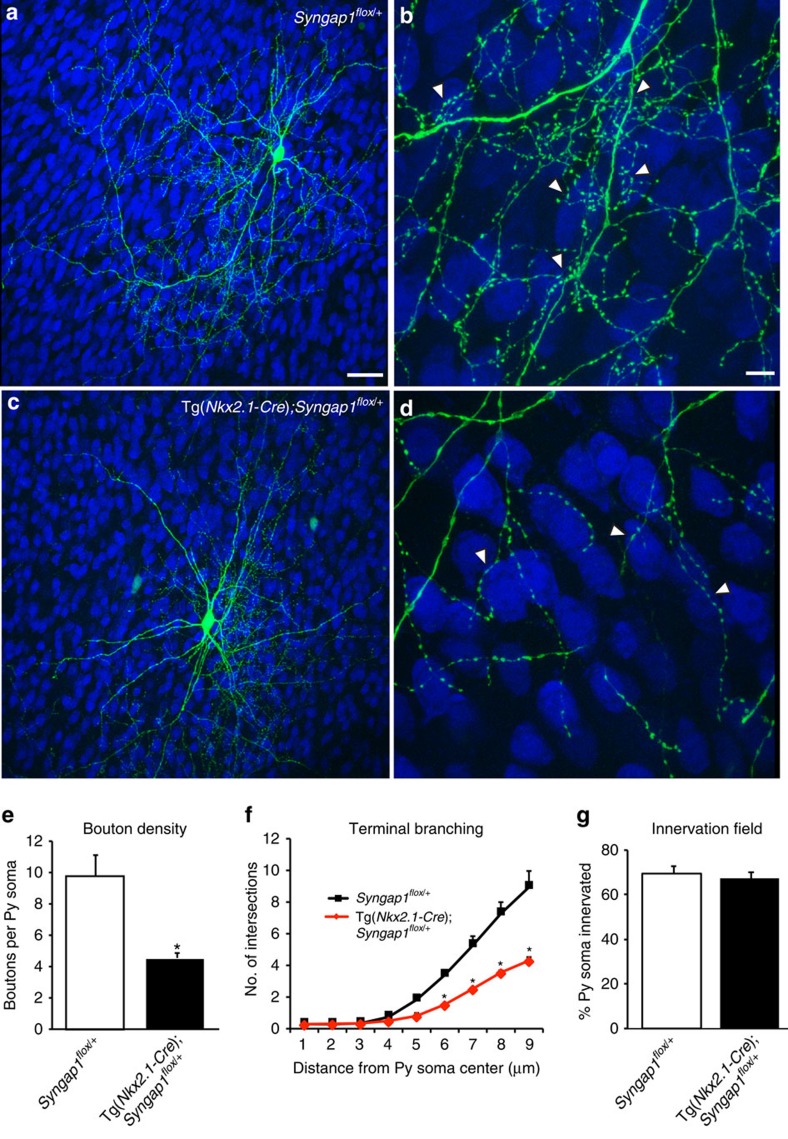
*Syngap1 *promotes terminal axonal branching and bouton formation in cortical MGE-derived basket cells. (**a**) A GABAergic basket cell transfected with pGAD_67_-GFP (green) in EP24 cortical organotypic cultures from *Syngap1*^*flox/+*^ mice showing dense axonal innervation and clustered perisomatic boutons (**b**, arrowheads) around pyramidal somata (NeuN, blue). (**c**) In contrast, age-matched basket cells from organotypic cultures from Tg(*Nkx2.1-Cre*);*Syngap1*^*flox/+*^mice show strongly reduced terminal axonal branching and bouton density around neuronal somata (**d**, arrowheads). (**e**,**f**) MGE-derived basket cells from Tg(*Nkx2.1-Cre*);*Syngap1*^*flox/+*^show significantly reduced bouton density and less developed terminal branching (**e**: Student's *t*-test, *P=0.02; **f**: Student's *t*-test, *P*=0.035 at 6 μm, *P*=0.020 at 7 μm, *P*=0.045 at 8 μm and *P*=0.020 at 9 μm) compared with age-matched basket cells from *Syngap1*^*flox/+*^ cultures. (**g**) The percentage of innervated cells does not differ between the two phenotypes (Student's *t*-test, *P*=0.623), suggesting that *Syngap1* promotes the formation of local perisomatic innervations without affecting overall axonal growth. *n*=6 basket cells from 3 *Syngap1*^*flox/+*^mice; *n*=7 basket cells from 3 Tg(*Nkx2.1-Cre*);*Syngap1*^*flox/+*^ mice. Scale bar, (**a**,**c**) 50 μm; (**b**,**d**) 10 μm.

To further investigate how *Syngap1* haploinsufficiency in MGE-derived GABAergic in MGE-derived GABAergic cells affect the formation of affects the formation of PV+ basket cell axonal arbour and synaptic boutons, we prepared cortical organotypic cultures from P4 Tg(*Nkx2.1-Cre*);*Syngap1*^*flox/+*^ mice and their control littermates. PV+ basket cells were labelled by biolistic transfection of P_G67_-GFP. In EP24 organotypic cultures from control mice (*Syngap1*^*flox/*+^), NeuN-positive somata were surrounded by highly branched basket cell axonal terminals and numerous perisomatic boutons (Fig. 3a,b); on the contrary, in age-matched cultures from Tg(*Nkx2.1-Cre*);*Syngap1*^*flox/+*^ mice, we found a significant decrease of the complexity of the terminal axonal branching (Fig. 3c,d,f) as well as of the perisomatic bouton density formed by basket cell around targeted neurons (Fig. 3e; boutons/soma, 10±1 for *Syngap1*^*flox/+*^ versus 4.7±0.3 for Tg(*Nkx2.1-Cre*); *Syngap1*^*flox/+*^, Students *t*-test *P*=0.02). Similarly to what we observed in single-basket cell knockout of *Syngap1* performed at EP10-24, the fraction of innervated somata was not affected by *Syngap1* knockdown in MGE-derived PV+ basket cells at the embryonic stage (Fig. 3g, percentage of innervation, 69%±3% for *Syngap1*^*flox/+*^versus 67%±3% for Tg(*Nkx2.1-Cre*);*Syngap1*^*flox/+*^; Students *t*-test, *P*=0.623). All together, these data show that *Syngap1* haploinsufficiency in MGE-derived neuron affects the formation of PV+ basket cell innervations *in vivo*.

### MGE-specific Syngap1 knockdown impaired GABA transmission

**Figure 4:**
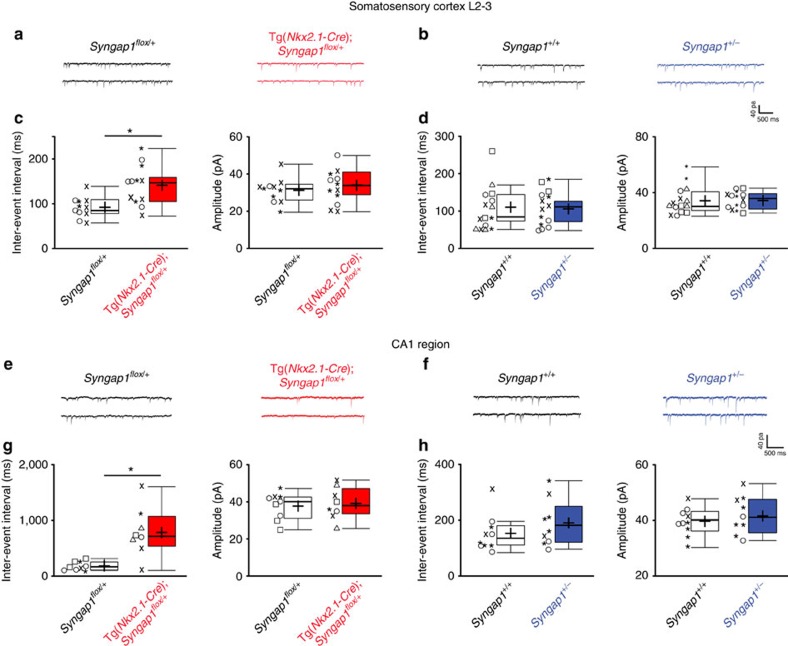
*Syngap1* haploinsufficiency in MGE-derived interneurons reduces mIPSC frequency. (**a**,**b**) Representative traces of mIPSCs from L2-3 pyramidal neurons in acute slices from *Syngap1*^*flox/+*^versus Tg(*Nkx2.1-Cre);Syngap1*^*flox/+*^ (**a**) and *Syngap1*^*+/+*^ versus* Syngap1*^*+/−*^ (**b**) young adult mice. (**c**,**d**) mIPSC inter-event intervals are significantly longer in Tg(*Nkx2.1-Cre*)*;Syngap1*^*flox/+*^compared with *Syngap1*^*flox/+*^ mice (**c**, LMM, genotype effect=−49.117, *P=0.004186), but not in *Syngap1*^*+/−*^ versus* Syngap1*^*+/+*^ mice (**d**, LMM, genotype effect=−1.952, *P*=0.5703). mIPSC amplitude is not affected in either mouse line (**c**,**d**; LMM, *P*>0.5). (**e**,**f**) Representative traces of mIPSCs from CA1 pyramidal neurons in acute slices from *Syngap1*^*flox/+*^versus Tg(*Nkx2.1-Cre);Syngap1*^*flox/+*^ (**e**) and *Syngap1*^*+/−*^ versus* Syngap1*^*+/+*^ (**f**) young adult mice. (**g**,**h**) mIPSC inter-event intervals are significantly increased in Tg(*Nkx2.1-Cre*)*;Syngap1*^*flox/+*^compared with *Syngap1*^*flox/+*^ mice (**g**; LMM, genotype effect=−605.15, *P=0.00109 for IEI), but they are not significantly affected in neurons from *Syngap1*^*+/−*^mice compared with control littermates (**h**; LMM, genotype effect=0.1203, *P*=0.9974 for IEI). mIPSC amplitude is not affected in either mouse line (LMM, *P*>0.5). mIPSC frequency and amplitude are represented with Tukey boxplots (median±1.5 × interquartile range; box represents 1st–3rd quartile). The cross represents mean values. Individual cell values from the same animal are represented with the same symbols. Somatosensory cortex L2-3: *n*=11 neurons from *n*=3 *Syngap1*^*flox/+*^ mice; *n*=12 neurons from *n*=3 Tg(*Nkx2.1-Cre*);*Syngap1*^*flox/+*^mice; *n*=15 neurons from *n*=6 *Syngap1*^*+/+*^ mice; *n*=14 neurons from *n*=4 *Syngap1*^*+/−*^. CA1: *n*=9 neurons from *n*=4 *Syngap1*^*flox/+*^ mice; *n*=8 neurons from *n*=5 Tg(*Nkx2.1-Cre*);*Syngap1*^*flox/+*^mice; *n*=10 neurons from *n*=3 *Syngap1*^*+/+*^ mice; *n*=9 neurons from *n*=3 *Syngap*^*+/−*^ mice.

The observation that PV+ basket cells in Tg(*Nkx2.1-Cre*);*Syngap1*^*flox/+*^ formed less boutons around their potential postsynaptic targets raises the possibility of an impairment in GABAergic synaptic transmission. Thus, we examined the effects of MGE-specific *Syngap1* knockdown on spontaneous inhibitory synaptic transmission in acute cortical and hippocampal slices from adult Tg(*Nkx2.1-Cre*);*Syngap1*^*flox/+*^mice and their respective control littermates. We found that mean inter-event interval (IEI) of miniature inhibitory postsynaptic currents (mIPSCs) recorded from pyramidal neurons in L2-3 somatosensory cortex was significantly longer in Tg(*Nkx2.1-Cre*);*Syngap1*^*flox/+*^mice relative to the controls (Fig. 4a,c; IEI: 92±7 ms for *Syngap1*^*flox/+*^ versus 143±17 ms Tg(*Nkx2.1-Cre*);*Syngap1*^*flox/+*^; linear mixed model (LMM), *P*=0.004186). Similarly, inter-event intervals of mIPSCs recorded from CA1 pyramidal neurons were significantly longer in Tg(*Nkx2.1-Cre*);*Syngap1*^*flox/+*^mice relative to the controls (Fig. 4e,g; IEI: 184±26 ms for *Syngap1*^*flox/+*^ versus 789±157 ms Tg(*Nkx2.1-Cre*);*Syngap1*^*flox/+*^; LMM, *P*=0.00109). Amplitude of mIPSCs was not affected in Tg(*Nkx2.1-Cre*);*Syngap1*^*flox/+*^mice compared with controls (Fig. 4a,c,e,g; LMM, *P*>0.5). Finally, mIPSC kinetics and mIPSC charge transfer were not significantly different for cortical and hippocampal neurons between the different genotypes (Supplementary Fig. 4). Therefore, *Syngap1* haploinsufficiency in MGE-derived interneurons *in vivo* strongly affects GABAergic transmission in adult mice.

To investigate whether inhibitory neurotransmission is also affected by germ-line deletion of *Syngap1,* we performed the same analysis in *Syngap1*^*+/−*^ and control littermates. We found no difference in inter-event intervals of mIPSC recorded from either L2-3 or CA1 pyramidal neurons in *Syngap1*^*+/−*^ compared with control mice (Fig. 4b,d,f,h; L2-3: IEI: 111±15 ms for *Syngap1*^*+/+*^ versus 106±11 ms for *Syngap1*^*+/−*^; CA1: IEI: 153±21 ms for *Syngap1*^*+/+*^ versus 191±28 ms for *Syngap1*^*+/−*^; LMM, *P*>0.5). mIPSC amplitude, kinetics and charge transfer were also not affected (Fig. 4b,d,f,h and Supplementary Fig. 4). The differences observed between *Syngap1*^*+/−*^ and Tg(*Nkx2.1-Cre*);*Syngap1*^*flox/+*^ mice suggest that the concurrent *Syngap1* haploinsufficiency in glutamatergic and/or in GABAergic neurons, which do not originate from the MGE alters neuronal circuit development in a manner that compensates, at least partly, inhibitory synaptic deficits originating from *Syngap1* haploinsufficiency in MGE-derived GABAergic neurons.

**Figure 5:**
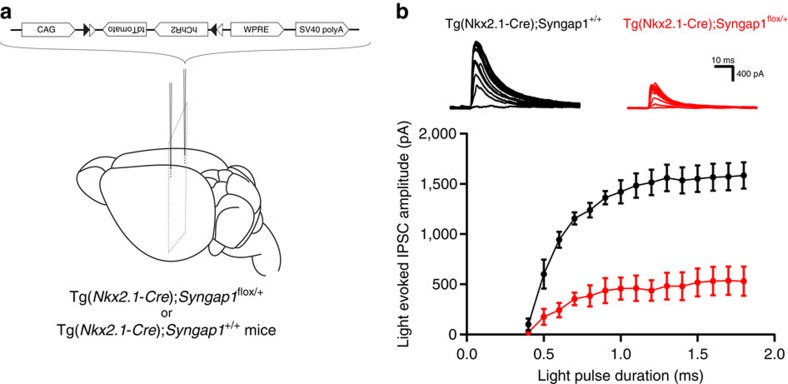
*Syngap1* haploinsufficiency in MGE-derived interneurons reduces their synaptic output to pyramidal cells. (**a**) Schematic of the experimental approach. pAAV.CAGGS.flex.ChR2.tdTomato.SV40 is bilaterally injected in somatosensory cortex of either Tg(*Nkx2.1-Cre*);*Syngap1*^*flox/+*^ or Tg(*Nkx2.1-Cre*);*Syngap1*^*+/+*^ young adult mice. This strategy allows ChR2 and tdTomato expression only in cells expressing Cre. (**b**) Upper panel. Traces of IPSCs recorded in response to light pulses of different duration in representative somatosensory cortex layer 5 pyramidal neurons in acute slices from Tg(*Nkx2.1-Cre*);*Syngap1*^*+/+*^ (black) and Tg(*Nkx2.1-Cre*);*Syngap1*^*flox/+*^ (red) mice. Lower panel. Summary graph showing data from input-output function of inhibitory responses evoked by light stimulation. The IPSC amplitudes are significantly lower in Tg(*Nkx2.1-Cre*);*Syngap1*^*flox/+*^ compared with Tg(*Nkx2.1-Cre*);*Syngap1*^*+/+*^, starting from the third point of the input-output function (two-way Repeated Measure ANOVA with Bonferroni's multiple comparison *post hoc* test, *P*<0.0001). *n*=4 Tg(*Nkx2.1-Cre*);*Syngap1*^*+/+*^mice (7 neurons) and *n*=4 Tg(*Nkx2.1-Cre*);*Syngap1*^*flox/+*^ mice (9 neurons).

To further confirm that *Syngap1* haploinsufficiency in MGE-derived interneurons impairs functional cortical GABAergic connectivity *in vivo*, we targeted expression of the light-sensitive channel channelrodopsin-2 (ChR2) to MGE-derived GABAergic cells, by injecting a Cre-dependent adeno-associated virus in somatosensory cortex^47^ (Fig. 5a). Two-weeks post-transfection, we recorded inhibitory synaptic responses evoked by blue light pulses of different duration (input-output function) in layer 5 pyramidal cells. The amplitude of light-evoked IPSCs was significantly smaller in Tg(*Nkx2.1-Cre*);*Syngap1*^*flox/+*^ mice compared with those recorded in Tg(*Nkx2.1-Cre*);*Syngap1*^*+/+*^ littermates (Fig. 5b; two-way Repeated Measure ANOVA with Bonferroni's multiple comparison *post hoc* test, *P*<0.0001). Together with the observation that the number of cortical perisomatic boutons formed by PV+ baskets was reduced in Tg(*Nkx2.1-Cre*);*Syngap1*^*flox/+*^ (Figs 2 and 3), this result demonstrated that *Syngap1* haploinsufficiency impaired GABAergic synapse formation and function of MGE-derived interneurons.

### MGE-specific Syngap1 knockdown affected gamma oscillations

PV+ cells represent the large majority of cortical GABAergic interneurons generated by MGE-derived precursors. Correlative, causal and computational evidence indicates that gamma oscillations in the 30–80 Hz range depend on synchronous activity of PV+ cells.^48^,^49^ Since we found that PV+ cell connectivity was reduced in both cortex and hippocampus of Tg(*Nkx2.1-Cre*);*Syngap1*^*flox/+*^ mice (Fig. 2), we hypothesized that PV+ cell synaptic impairment would lead to changes in gamma oscillatory activity in these mice.

**Figure 6:**
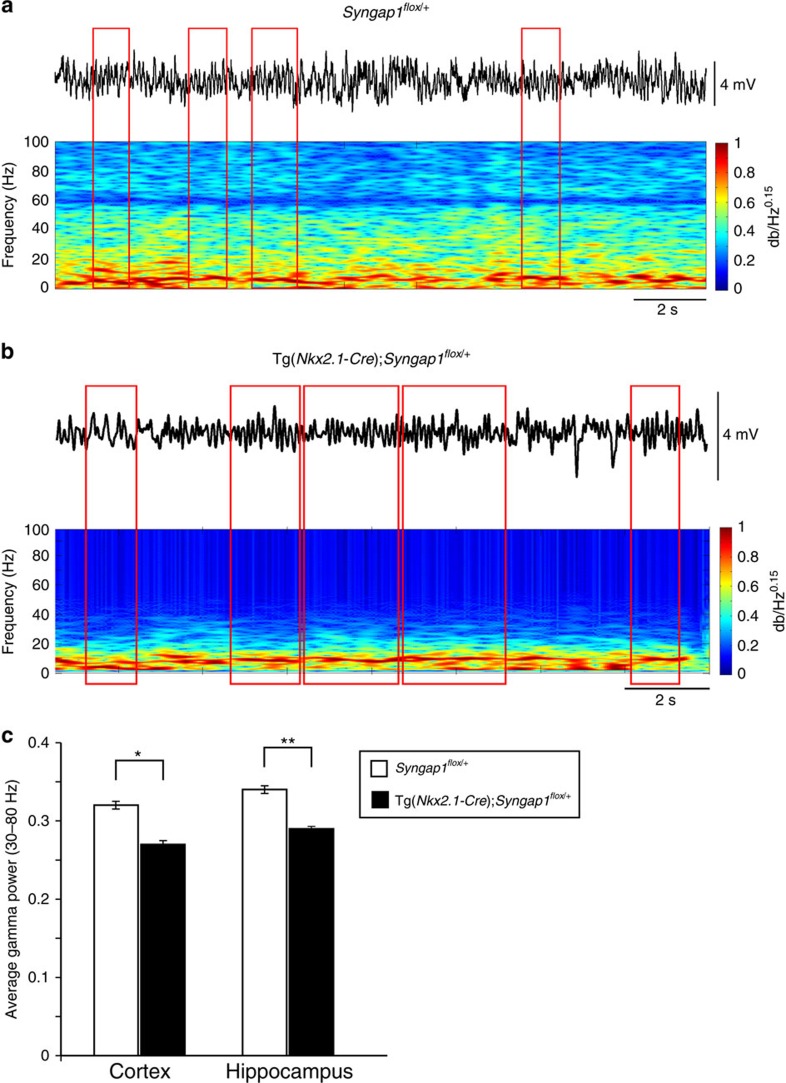
*Syngap1* haploinsufficiency in MGE-derived interneurons impairs cortical gamma oscillations during active exploration in awake mice. (**a**,**b**) Representative examples of EEG recordings from the cortex of adult (**a**) *Syngap1*^*flox/+*^ and (**b**) Tg(*Nkx2.1-Cre*);*Syngap1*^*flox/+*^mice with corresponding spectrogram. Note that although theta oscillations (red boxes) are detected in both animals, gamma power is lower in Tg(*Nkx2.1-Cre*);*Syngap1*^*flox/+*^during these oscillations. (**c**) Bar graph showing the average power in the gamma frequency band in the cortex and the hippocampus for both groups. Note that in both regions, Tg(*Nkx2.1-Cre*);*Syngap1*^*flox/+*^ showed less power in the gamma frequency band (Wilcoxon rank sum tests, *P=0.005, **P=0.0006). *n*=3 mice for both genotype.

To test this hypothesis, we measured specific oscillatory patterns by video-electroencephalogram (EEG) recordings during active exploratory behaviour. We found that in the cortex, average power in the gamma frequency band was significantly lower in Tg(*Nkx2.1-Cre*);*Syngap1*^*flox/+*^ animals compared with control littermates (Fig. 6; 0.27±0.02 versus 0.32±0.02 for Tg(*Nkx2.1-Cre*);*Syngap1*^*flox/+*^ and *Syngap1*^*flox/+*^, respectively; Wilcoxon rank sum test, *P*=0.01). Similar results were observed in the hippocampus (Fig. 6c; 0.29±0.02 versus 0.335±0.005 for Tg(*Nkx2.1-Cre*);*Syngap1*^*flox/+*^ and *Syngap1*^*flox/+*^, respectively; Wilcoxon rank sum test *P*=0.001). Taken together, these data demonstrate that *Syngap1* haploinsufficiency in MGE-derived interneurons alters brain network activity, which in turn may contribute to cognitive deficits.

### MGE-specific Syngap1 knockdown caused cognitive deficits

While *Syngap1*^*−/−*^ mice die within the first days after birth, adult *Syngap1*^*+/−*^ mice showed behavioural and cognitive deficits, including hyperactivity, anxiolysis, abnormal reference, working and social memories^12^,^14^,^15^,^16^,^17^. We have previously proposed that *Syngap1*^*+/−*^mice represent an appropriate model to study the cognitive and behavioural abnormalities associated with the disruption of *SYNGAP1* in humans, as pathogenic mutations documented up to now in this gene are predicted to cause a loss-of-function.^7^

**Figure 7:**
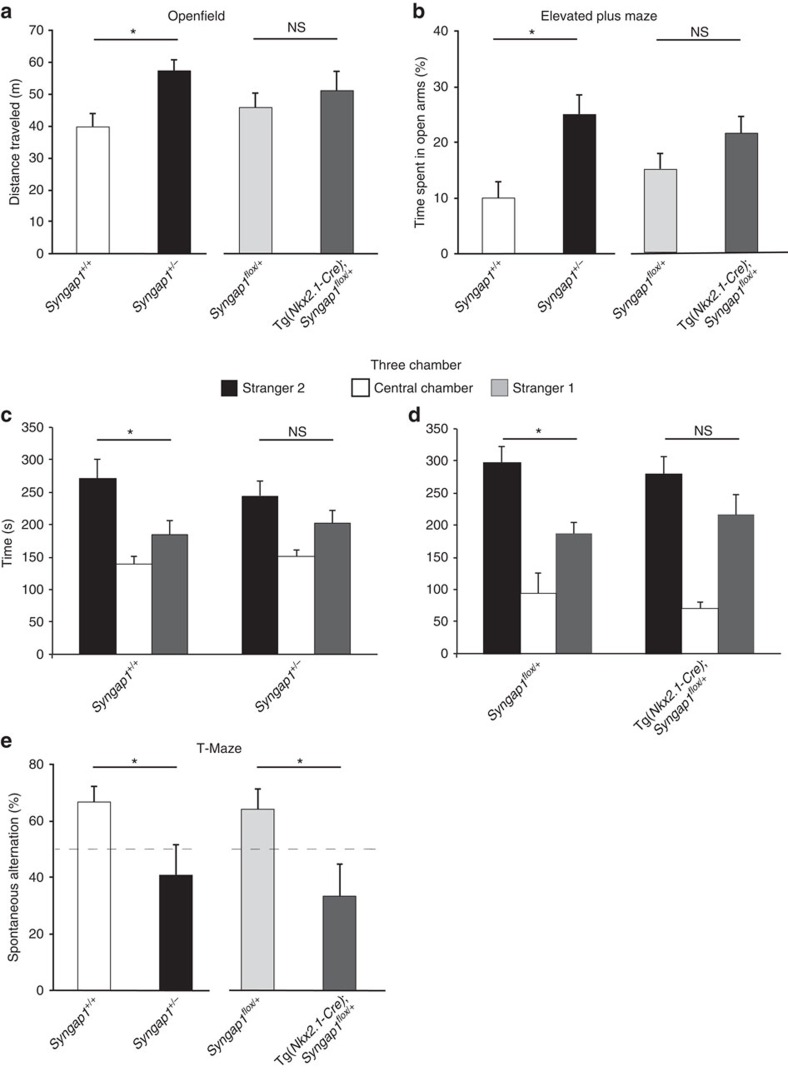
Tg(*Nkx2.1-Cre*);*Syngap1*^*flox/+*^ mice show cognitive deficits that partially recapitulate those observed in *Syngap1*^*+/−*^mice. (**a**,**b**) *Syngap1*^*+/−*^mice show hyperactivity (**a**; Mann–Whitney rank sum test, *P=0.0032) and altered stress levels (**b**; Student'st -test, *P=0.01), while Tg(*Nkx2.1-Cre*);*Syngap1*^*flox/+*^ mice behave similarly to their control littermates in the open field (**a**) and elevated plus maze (**b**) tests (**a**; Mann–Whitney rank sum test, *P*=0.503, **b**: Student's *t*-test, *P*=0.136; NS, not significant). (**c**,**d**) *Syngap1*^*+/−*^mice (**c**) and Tg(*Nkx2.1-Cre*);*Syngap1*^*flox/+*^ (**d**) mice show social novelty preference impairment and spend similar amount of time in the chambers with either the novel (Stranger 2) or the familiar mouse (stranger 1), contrary to their control littermates, which spend more time with the novel mouse (Stranger 2 × Stranger 1 × Genotype, Two-way ANOVA with Sidak's multiple comparison *post hoc* test, *P<0.05). (**e**) Both Tg(*Nkx2.1-Cre*);*Syngap1*^*flox/+*^ and *Syngap1*^*+/−*^mice perform worse than their littermates on the T-maze test (Student's *t*-test, *P=0.035 for *Syngap1*^*+/−*^versus *Syngap1*^*+/+*^ and *P=0.038 for Tg(*Nkx2.1-Cre*);*Syngap1*^*flox/+*^ versus *Syngap1*^*flox/+*^). Open field and Elevated T-Maze: *n*=12 *Syngap1*^*+/+*^, *n*=11 *Syngap1*^*+/−*^, *n*=12 *Syngap1*^*flox/+*^, *n*=11 Tg(*Nkx2.1-Cre*);*Syngap1*^*flox*/+^ mice. For three-chamber test: *n*=10 *Syngap1*^*+/+*^, *n*=10 *Syngap1*^*+/−*^, *n*=10 *Syngap1*^*flox/+*^, *n*=16 Tg(*Nkx2.1-Cre*);*Syngap1*^*flox*/+^ mice. For T-maze, *n*=15 *Syngap1*^*+/+*^, *n*=9 *Syngap1*^*+/−*^, *n*=13 *Syngap1*^*flox/+*^ *n*=9 Tg(*Nkx2.1-Cre*);*Syngap1*^*flox/+*^mice.

To understand to what extent *Syngap1* haploinsufficiency in MGE-derived GABAergic neurons may contribute to the cognitive and behavioural deficits observed in *Syngap1*^*+/−*^ mice, we compared the performance of young adult *Syngap1*^*+/−*^, Tg(*Nkx2.1-Cre*);*Syngap1*^*flox/+*^mice and their respective control littermates in several behavioural tests. We first assessed the locomotion activity using the open-field arena. Relative to their wild-type littermates, *Syngap1*^*+/−*^ mice travelled more during a 10 min session (Fig. 7a; 40±4 m for *Syngap1*^*+/+*^ versus 57±6 m for *Syngap1*^*+/−*^, Mann–Whitney rank-sum test, *P*=0.0032), which is consistent with previously published observations^12^,^14^,^15^,^16^. These results indicate that *Syngap1*^*+/−*^ mice are hyperactive and that they do not display major locomotor deficits. Next, we tested anxiety-like behaviour using the elevated plus-maze test. *Syngap1*^*+/−*^ mice spent significantly more time in the open arms than the wild-type littermates (Fig. 7b; 10±2% for *Syngap1*^*+/+*^ versus 25±4% for *Syngap1*^*+/−*^, Student's *t*-test, *P*=0.01), suggesting a reduced level of anxiety-like behaviour in the germ-line *Syngap1* heterozygous mice, as reported in previous studies^14^,^16^. Conversely, Tg(*Nkx2.1-Cre*);*Syngap1*^*flox/+*^ showed normal levels of locomotion and anxiety-like behaviour compared with *Syngap1*^*flox/+*^mice (Fig. 7a,b; for open field: 45±5 m for *Syngap1*^*flox/+*^ versus 51±6 m for Tg(*Nkx2.1-Cre*);*Syngap1*^*flox*^/+, Mann–Whitney rank sum test, *P*=0.503; for elevated plus maze: 15±3% for *Syngap1*^*flox*/+^ versus 21±3% Tg*(Nkx2.1-Cre);Syngap1*^*flox/+*^, Student's *t*-test, *P*=0.136).

Several mouse models of ID and autism exhibit reduced social interaction behaviour^17^,^23^. We tested social interactions in *Syngap1*^*+/−*^ and Tg(*Nkx2.1-Cre*);*Syngap1*^*flox/+*^ and their respective littermates. After a habituation period, we evaluated sociability by assessing the preference of the tested mice for an unknown wild-type conspecific mouse matched for age, sex and background versus an empty wire cage (Supplementary Fig. 5). Mice of all genotypes spent significantly more time with an unfamiliar mouse (stranger 1) relative to the empty wire cage (Supplementary Fig. 5, two-way ANOVA *P*<0.05), indicating that social approach behaviour was normal in *Syngap1*^*+/−*^ and Tg(*Nkx2.1-Cre*);*Syngap1*^*flox/+*^ relative to their respective control littermates. Next, we tested whether social novelty preference was affected in these mice by assessing the preference for a second stranger mouse. *Syngap1*^*+/+*^ and *Syngap1*^*flox/+*^ mice spent significantly more time in the chamber containing the novel mouse (stranger 2) than in the chamber with the familiar one (stranger 1; Fig. 7c,d; stranger 2 vs stranger 1 (in s): 271±29 versus 184±22 for *Syngap1*^*+/+*^; 297±27 versus 187±18 for *Syngap1*^*flox/+*^; two-way ANOVA *post hoc* Sidak's multiple comparisons test *P*<0.05). In contrast, both *Syngap1*^*+/−*^ and Tg(*Nkx2.1-Cre*);*Syngap1*^*flox/+*^ spent similar amount of time with the novel versus the familiar mice (Fig. 7c,d, stranger 2 versus stranger 1 (in s): 245±23 versus 203±20 for *Syngap1*^*+/−*^; 279±26 versus 216±32 for Tg(*Nkx2.1-Cre*);*Syngap1*^*flox/+*^; two-way ANOVA *post hoc* Sidak's multiple comparisons test *P*>0.05), indicating deficits in social novelty preference in both *Syngap1*^*+/−*^ and Tg(*Nkx2.1-Cre*);*Syngap1*^*flox/+*^ mice.

Finally, we tested spatial working memory by measuring spontaneous alternation in the T-maze. Spontaneous alternation was strongly altered in both *Syngap1*^*+/−*^ and Tg(*Nkx2.1-Cre*);*Syngap1*^*flox/+*^ relative to their respective control littermates (Fig. 7e; 67±6% for *Syngap1*^*+/+*^ versus 41±11% for *Syngap1*^*+/−*^, Student's *t*-test *P*=0.035, and 64±7% for *Syngap1*^*flox/+*^ versus 33.333±11% for Tg(*Nkx2.1-Cre*);*Syngap1*^*flox/+*^, Student's *t*-test *P*=0.038), suggesting impaired spatial working memory in both mutant lines. On the other hand, we found that Tg(*Nkx2.1-Cre*) mice showed no statistical difference in locomotion, anxiety, spontaneous alternation and social recognition behaviour (Supplementary Fig. 6). All together, these data show that specific inactivation of *Syngap1* in MGE-derived interneurons recapitulates at least in part the behavioural and cognitive alterations observed in mice with germ-line *Syngap1* haploinsufficiency.

## Discussion

Our data show that *Syngap1* is required for the proper terminal axonal branching and bouton formation of cortical GABAergic PV+ basket cells, both in organotypic cortical cultures and *in vivo*. Further, MGE-restricted *Syngap1* haploinsufficiency causes deficits in cortical evoked-inhibitory synaptic transmission and gamma oscillations and specific impairments in social novelty preference and working memory, in adult mice. In contrast, a recent study did not observe any cognitive deficits in *Gad2-cre;Syngap1*^*flox*/+^ mice^17^. One possible explanation for this discrepancy is that *Nkx2.1* and *Gad2* may be expressed at different developmental time points. In fact, *Nkx2.1* is already expressed in all MGE-derived GABAergic interneurons by E10.5. (ref. 45) On the other hand, while *Gad2* transcription starts early during embryogenesis^50^ (around E11.5), it is possible that not all GABAergic cells express it at the same time. For example, GAD65, which is coded by *Gad2*, is barely expressed before P6 in rodent somatosensory cortex^51^. *Syngap1* haploinsufficiency appears to disrupt the function of excitatory circuits mainly by affecting their early synaptic development^16^,^17^. Our observations suggest that *Syngap1* is also required during the early phases of the development of GABAergic circuit connectivity.

Another major difference is that, while Gad2 is expressed by most GABAergic interneurons, Nkx2.1 is expressed only by interneurons derived from the MGE, which include PV+ and SST+ interneurons but not, for example, those positive for the vasoactive intestinal polypeptide (VIP). Recent results demonstrate that VIP-expressing neurons form a disinhibitory microcircuit that is conserved across cortical regions^52^. In particular, VIP interneurons tend to inhibit most SST+ and a fraction of PV+ interneurons. Inhibition of these interneurons in turn disinhibits principal cells *in vivo*, providing a form of gain control.^52^ It is possible that removing *Syngap1* in all interneurons may allow homeostatic synaptic adjustments that are not otherwise engaged when *Syngap1* is removed only in MGE-derived interneurons. It will therefore be important to assess whether and how *Syngap1* regulates the synaptic connectivity of distinct GABAergic interneuron types other than PV+ basket cells.

Disruption of excitatory/inhibitory balance is emerging as a common theme for many neurodevelopmental disorders^53^,^54^. However, the critical challenge is to understand how specific circuit alterations resulting from mutations affect the development of cognitive abilities. Our study suggests new avenues of research for the understanding of mechanisms involved in *SYNGAP1*-related ID. For example, our results showed that *Syngap1* plays a role in the formation of perisomatic synapses by PV+ basket cells. GABAergic neurotransmission from these cells promotes network gamma oscillations^48^,^55^. Cortical gamma oscillations increase in power during tasks that require complex processing of sensory information, attention, working memory and cognitive control, suggesting that gamma oscillations are crucial for cognition^56^. Interestingly, substantial evidence indicates that individuals with specific psychiatric disorders, such as schizophrenia or autism, show lower power of gamma oscillations induced during the performance of cognitive tasks compared with healthy individuals^57^,^58^. Here, we showed that MGE-specific *Syngap1* haploinsufficiency leads to reduced gamma oscillation power during exploration, which in turn may contribute to the occurrence of cognitive deficits. It will be interesting to explore the presence of alterations in brain oscillations in patients carrying *SYNGAP1* mutations. Furthermore, cortical GABAergic inhibition, in particular the one mediated by PV+ cells, has been suggested to play a key role in critical period plasticity in the developing brain^21^,^59^. Critical periods represent heightened epochs of brain plasticity during childhood, during which experience can produce permanent, large-scale changes in neuronal circuits. By regulating critical period plasticity, PV+ cell-mediated inhibition may influence how experience shapes brain wiring during early life. As a consequence, altered PV cell connectivity and function could drive cortical neuronal circuits along abnormal developmental trajectories, which could then contribute to cognitive dysfunction. Ultimately, the understanding of *SYNGAP1* function in specific inhibitory and excitatory circuits might lead to the development of tailored therapies.

MGE-derived interneurons include PV+ basket cells and SST+ interneurons^60^, thus alterations in the synaptic connectivity of this latter group may also contribute to the cognitive deficits that were observed in Tg(*Nkx2.1-Cre*);*Syngap1*^*flox/+*^ mice. Further, it is very likely that altered GABAergic signalling results in either causal or compensatory alterations in non-GABA circuits as well, which can further play a role in cognitive dysfunctions in mutant mice. The main challenges will be to understand when these alterations arise and whether and to what extent cognitive deficits are due to either altered neural circuit development leading to diverse changes in brain and behaviour and/or a state of continuous synaptic dysfunction in adulthood.

Haploinsufficiency of the *NF1* gene, which also codes for a RasGAP, causes neurofibromatosis type 1, a complex neuro-cutaneous condition that is associated with cognitive impairment and autism spectrum disorders. Decrease of *Nf1* induces deficits in hippocampal-dependent spatial learning, amygdala dependent-social learning and prefrontal cortex-dependent working memory by increasing GABA release in these different areas of the brain through the activation of the Ras–ERK pathway^61^,^62^,^63^,^64^. The increase of Ras thus appears to produce opposite effects on GABA neurotransmission depending upon whether it is associated with *Syngap1* or *Nf1* haploinsufficiency. How can we explain this paradox? One possibility is that *Syngap1* and *Nf1* affect Ras activity at different time points during the development of GABAergic circuits. As discussed above, *Syngap1* haploinsufficiency might impact the establishment of GABAergic connectivity by acting during early synaptic development. *Nf1* might on the other hand modulate GABA release in mature synapses. Alternatively, it is possible that SYNGAP1 and the *Nf1* product, neurofibromin, function in distinct subcellular compartments of GABAergic neurons and thus regulate different processes. SYNGAP1 is associated with NMDA receptor complexes at the postsynaptic membrane of glutamatergic neurons^1^,^2^,^29^, raising the possibility that it also interacts with such receptors in GABAergic neurons^65^. In contrast, neurofibromin has been shown to localize to smooth vesiculotubular elements, cisternal stacks and multivesicular bodies in the cell body and dendrites of Purkinje cells, but not with the plasma membrane^66^. All together, these observations suggest that SYNGAP1 and neurofibromin modulate the development and function of GABAergic cells by regulating differently the spatial and/or temporal activity of Ras.

In summary, our study suggests that *Syngap1* haploinsufficiency affects GABAergic circuit connectivity and function, which in turn may contribute to cognitive alterations. Further studies will be required to determine whether pharmacological interventions during development can rescue these deficits.

## Methods

### Mice

All procedures described here had been approved by the Comité Institutionnel de Bonnes Pratiques Animales en Recherche (CIBPAR) of the Research Center of Sainte-Justine Hospital in accordance with the principles published in the Canadian Council on Animal's Care's (Guide to the Care and Use of Experimental Animals, Feb 91). *Syngap1*^*+/−*^ mice were kindly provided by Dr Seth Grant (Edinburgh University, United Kingdom) and *Syngap1*^*flox/flox*^ by Drs. Irene Knuesel and Mary Muhia (University of Zurich, Switzerland). They were generated and genotyped as described in refs 10, 15. They were maintained as a heterozygous line onto a C57Bl/6 background. *Syngap1*^*flox/flox*^mice were crossed with a mouse line bearing a transgene encoding the enzyme Cre recombinase under the control of the Nkx2.1 promoter (Jackson Laboratory #008661). This promoter drives the expression of the Cre protein in GABAergic interneurons derived from the MGE starting at embryonic day 10.5 (ref. 45). The resulting animals were referred to as Tg(*Nkx2.1-Cre*)*;Syngap1*^*flox/+*^ mice. To obtain Tg(*Nkx2.1-Cre*)*;Syngap1*^*flox/flox*^ mice, we crossed Tg(*Nkx2.1-Cre*)*;Syngap1*^*flox/+*^ individuals with *Syngap1*^*flox/flox*^ mice. To validate the specificity of Cre expression, we backcrossed these mice with a GFP reporter line (RCE mouse^46^). Animals of both sexes were used in all the experiments.

### Primary cortical neuron culture

Cortical neurons from E18 rat embryos were dissociated mechanically and plated on dishes treated with poly-D-lysine (0.1 mg ml^−1^; Sigma-Aldrich, St. Louis, MO). Neurons were cultured overnight in attachment medium: MEM (Invitrogen) supplemented with 1 mM sodium pyruvate (Invitrogen), 0.6% D-glucose (Sigma-Aldrich) and 10% horse serum. The medium was replaced the next day with maintenance medium: Neurobasal-A medium (Invitrogen) supplemented with 2% B27 (Invitrogen) and 1% L-glutamine (Invitrogen). The maintenance medium was replaced with fresh medium at 3DIV. Subsequently, the medium was replaced every 6 days with maintenance medium until collecting the cells. Cultures were incubated in a humidified incubator at 37 °C with a 5% CO_2_-enriched atmosphere during 21 days then were fixed in 100% methanol (−20 °C) for 5 min. The fixation was stopped by adding 0.3 M glycine for 5 min. Neurons were permeabilized with 0.25% Triton in phosphate buffer pH 7.4, washed and blocked for 0.5 h in 3% BSA at room temperature. Cultures were then incubated for 1 h with anti-GAD67 antibody (mouse, 1:200, Abcam #26116) or anti-PV antibody (mouse, 1:500, Sigma, #P3088) and anti-SYNGAP1 antibody (rabbit, 1:200, Abcam #3344), followed by incubation with Alexa 488-conjugated IgG (goat anti-rabbit, 1:400, Invitrogen #A-11008) and Alexa 555-conjugated IgG (goat anti-mouse1:400, Invitrogen #A-21422) for 0.5 h at room temperature (RT), then mounted in Prolong (Invitrogen #P-7481) and stored at 4 °C. Images were acquired using a confocal microscope (LEICA TCS SPE or Leica TSC SP8).

### Cortical organotypic culture and biolistic transfection

Slice culture preparation and gene gun transfection were performed as described in^37^,^40^. Briefly, brains from postnatal days 4–5 *Syngap1*^*flox/flox*^, Tg(*Nkx2.1-Cre*)*;Syngap1*^*flox/+*^and *Syngap1*^*flox/+*^ were sliced coronally (400 μm thick) using a chopper (Stoelting). Slices were plated on Millicell membrane inserts. Culture medium (containing DMEM, 20% horse serum, 1 mM glutamine, 13 mM glucose, 1 mM CaCl_2_, 2 mM MgSO_4_, 0.5 μM ml^−1^ insulin, 30 mM HEPES, 5 mM NaHCO_3_, and 0.001% ascorbic acid) was placed under the membrane inserts. Slices were incubated in a humidified incubator at 34 °C with 5% CO_2_. Medium was changed three times per week. For biolisitic transfection, 1.6 μm gold particles (BioRad) were coated with 25–30 μg of each one of the plasmids of interest. DNA-coated gold particle were then shot using a gene gun (Bio-Rad) at high pressure (180ψ) at the age specified in the result section, incubated for 8 or 14 days in the same culture medium as described above. In experiments where *Syngap1* was deleted in PV+ single-basket cells in an otherwise wild-type background, organotypic slices from *Syngap1*^*flox/flox*^ mice were transfected either with pGAD_67_-*GFP (Syngap1*^*+/+*^, control basket cells), or pGAD_67_-*Cre*/pGAD_67_-*GFP* (*Syngap1*^*−/−*^ basket cells). In particular, for each mouse half of the organotypic cultures were transfected with pGAD_67_-*GFP* and half with pGAD_67_-*Cre*/pGAD_67_-*GFP*, thus allowing us to control for inter-animal variability. Organotypic slices from Tg(*Nkx2.1-Cre*)*;Syngap1*^*flox/+*^and *Syngap1*^*flox/+*^ were transfected with pGAD_67_-*GFP* to visualize PV+ basket cells morphology a minimum of 5 days before imaging.

### Analysis of basket cells *in vitro*

Slices were fixed and immunostained as in ref. 40. To visualize basket cell targeted neurons, we used anti-NeuN (monoclonal antibody, 1:400; Millipore Bioscience Research Reagents, #MAB377), followed by incubation with Alexa Fluor 633-conjugated goat anti-mouse IgG (1:400; Invitrogen A-21053). Only one basket cell was acquired from each organotypic culture. Non-overlapping fields of axonal innervation from well-isolated basket cells were acquired with a × 63 glycerol-immersion objective (NA 1.3; Leica) using a confocal microscope (LEICA TCS SPE) and them analyzed with Neurolucida software (MicroBrightField). Axon branch complexity around a single pyramidal cell soma was quantified as the average number of intersections between a basket cell axon and each one of 9 Sholl spheres traced with 1 μm increment from the centre of the pyramidal cell soma. Bouton density around each pyramidal cell soma was defined as the total number of GFP+ boutons identified in a radius of 9 μm from the centre of the pyramidal cell soma. For each basket cell, we analyzed 15–20 pyramidal neurons. The percentage of pyramidal somata innervated by basket cells was defined in a confocal stack by the number of somata contacted by the GFP+ axon divided by the total number of somata. At least two fields were imaged for each basket cell and the results were averaged. For these experiments, the number of basket cells was used as independent replicate (N). Data are presented as mean ±s.e.m. Imaging and quantification were performed independently by two different operators. All quantification was done blind to the genotype or the experimental conditions.

### Analysis of perisomatic innervation in vivo

P45 mice were anesthetized and perfused transcardially with 4% paraformaldehyde (PFA 4%) in phosphate buffer, pH 7.4. Brains were removed, immerged in PFA 4% overnight then cryoprotected in 30% sucrose (in sodium–phosphate buffer pH 7.2) and finally embedded in optimal cutting temperature Tissue Tek. Coronal sections (40 μm) from *Syngap1*^*+/−*^, Tg(*Nkx2.1-Cre*)*;Syngap1*^*flox/+*^ and their control littermates of both sexes were cut using a cryostat (Leica VT100). Brain sections were blocked in 10% normal goat serum (NGS) and 0.3% Triton X-100 for 2 h at RT. Slices were then incubated overnight at 4 °C with anti-Parvalbumin (rabbit 1:8,000, Swant, #PV25) and anti-NeuN antibodies (mouse 1:400, Chemicon, #MAB377) followed by incubation with Alexa 488-conjugated anti-rabbit IgG and Alexa 633-conjugated anti-mouse IgG (1:400, Invitrogen, A-11008 and A-21053, respectively) for 2 h at RT. After washing, slices were mounted in Vectashield mounting medium (Vector). Confocal images were taken using a × 63 oil objective (Leica NA 1.3). Scans from each channel were collected in multiple-track mode and subsequently merged. For each animal, three to four coronal sections containing the somatosensory cortex and an equal number containing the CA1 region of the hippocampus were imaged. Stacks were acquired with 1 μm steps, exported as TIFF files and analyzed with ImageJ software. We analyzed the intensity of PV-positive puncta around layer 5 pyramidal cell somata because the size of their apical dendrite rendered their identification fairly easy, therefore reducing the variability in the analysis. For each identified neuronal soma, intensity was quantified in the optical section where the soma profile was larger, in particular an area 2 μm distal from the edge of a neuron profile was traced and outlined, and staining intensity within this area was measured. Results of at least 30 NeuN-positive somata from the somatosensory cortex of each mouse were averaged. For these experiments, the number of animals was used as independent replicate (N). Data are presented as mean ±s.e.m. Imaging and quantification were performed independently by two different operators. All quantification was done blind to the genotype.

### Viral vector and stereotaxic injections

The pAAV.CAGGS.flex.ChR2.tdTomato.SV40 was a gift from Scott Sternson (Addgene plasmid # 18917) and was produced as AAV2/9 serotype by the UPenn Vector Core Facilities (University of Pennsylvania, Philadelphia). Mice aged postnatal day 24–28 (P24-P28) were used for all surgeries. Bilateral viral injections were performed at the following stereotaxic coordinates: 1.5 mm from bregma, 1.5 mm lateral from midline and 0.70 mm vertical from cortical surface. Surgical procedures were standardized to minimize the variability of AAV injections. To ensure minimal leak into surrounding brain areas, injection pipettes remained in the brain for ∼5 min after injection before being slowly withdrawn. The final injected volume for the AAV was 0.5 μl. We waited 2 weeks for maximal viral expression. The titre for the viruses was ∼2.69 × 10^12^ genome copies per ml.

### Electrophysiology

All the following experiments were done by investigators blind to the genotype until after data analysis was completed.

#### mIPSC recordings:

Acute slices (300 μm thickness) were prepared from hippocampus or somatosensory cortex of 7–9 week old *Syngap1*^*+/−*^, Tg(*Nkx2.1-Cre*)*;Syngap1*^*flox/+*^ mice and their respective control littermates of both sexes^25^. Slices were cut on a Leica vibratome in ice-cold oxygenated (95% O_2_, 5% CO_2_) cutting solution containing 87 mM NaCl, 2.5 mM KCl, 25 mM NaHCO_3_, 0.5 mM CaCl_2,_ 1.25 mM NaH_2_PO_4_, 7 mM MgCl_2_, 25 mM glucose, 75 mM sucrose, 3 mM pyruvic acid and 1 mM ascorbic acid. Slices were then transfered to oxygenated (95% O_2_, 5% CO_2_) artificial cerebrospinal fluid containing: 124 mM NaCl, 2.5 mM KCl, 1.25 mM NaH_2_PO_4_, 2 mM MgSO_4_, 2 mM CaCl_2_, 26 mM NaHCO_3_ and 10 mM glucose (pH 7.2–7.3; 300 mOsm) and allowed to recover at room temperature for at least 1 h before recording in artificial cerebrospinal fluid. Whole-cell patch clamp recordings were obtained from visually identified pyramidal neurons in hippocampal CA1 region or somatosensory cortex layer 2–3 using borosilicate pipettes (3–5 MΩ) and an upright microscope (Nikon Eclipse, E600FN), equipped with a water immersion long-working distance objective ( × 40, Nomarski optics) and an infrared video camera. To record mIPSCs, the internal solution contained 130 mM CsCl, 10 mM HEPES, 2 mM MgCl_2_, 0.5 mM EGTA, 5 mM di-Na-phosphocreatine, 2 mM ATP-Tris, 0.4 mM GTP-Tris, 5 mM QX-134 (pH 7.2–7.3; 280 mOsm; Alomone Labs). mIPSCs were measured by holding the cells at −60 mV in the presence of 10 μM CNQX, 50 μM D-APV and 0.5 μM TTX (to block AMPA receptors, NMDA receptors and sodium channels, respectively; Abcam Biochemicals). Data was acquired using Multiclamp 700A/B amplifiers (Molecular Devices) and digitized using Digidata 1322A/1440A and pClamp 10 (Molecular Devices). Recordings were low-pass filtered at 3 kHz and digitized at 20 kHz. Series resistance was regularly monitored during experiments and data were included only if the holding current and series resistance were stable. mPSCs were analysed using Mini-Analysis 6.0.3 (Synaptosoft) after detection of a well-defined baseline. Averages of at least 150 events from each cell were considered for analysis. Data are presented as mean ±s.e.m., unless otherwise specified.

#### Optogenetics:

Slices were prepared as described above. Whole-cell patch clamp recordings were obtained from visually identified pyramidal neurons in somatosensory cortex layer 5 using borosilicate pipettes (3–5 MΩ) and an upright microscope (Olympus BX50WI), equipped with a water immersion long-working distance objective ( × 40, Nomarski optics) and an infrared video camera. Care was taken to record from brain slices showing similar dense AAV tranfection, that is, tdTomato expression. The internal solution contained 120 mM CsMeSO3, 5 mM NaCl, 1 mM MgCl_2_, 10 mM HEPES, 10 mM diNa-phosphocreatine, 2 mM ATP-tris, 0.4 mM GTP-tris, 0.5 mM spermine and 2 mM QX-314-Cl. Light-evoked IPSCs were recorded by holding the cell at 0 mV in the presence of 10 μM CNQX and 50 μM DL-APV. IPSCs were evoked by brief pulses of blue light (470 nm, custom-made LED system) delivered to the whole slice every 30 s via a light guide positioned above the slice. Data was acquired using a Multiclamp 700A amplifier (Molecular Devices) and digitized using Digidata1440A and pClamp 10 (Molecular Devices). Recordings were low-pass filtered at 3 kHz and digitized at 20 kHz. Series resistance was regularly monitored during experiments, and data were included only if the holding current and series resistance were stable. Light-evoked IPSCs were analyzed using Clampfit 10 (Molecular devices). For statistical analysis, the number of animals was used as independent replicates (N).

#### EEG recordings and gamma oscillation analysis:

To analyze power in the gamma frequency band (30–80 Hz), we monitored P80–P100 mice by continuous video-EEG recordings after implantation of electrodes in the hippocampus and the overlying cortex. The insertion of electrodes was performed under isoflurane anaesthesia, while the head of the mouse was immobilized in a stereotaxic frame. A stainless steel bipolar electrode (Plastics-1 Inc., Roanoke, VA, USA) was positioned at the following coordinates with reference to Bregma: AP=−1 mm, ML=−1 mm and DV=1.5 mm for one electrode in the hippocampus and 0.5 mm for the one in the cortex. Following a 5-day recovery period, EEG was recorded with a Stellate systems 32-channel video-EEG recording unit for 2 weeks. Mice were monitored during 6-hour sessions (afternoon and night). An operator blind to the genotype extracted EEG traces during mouse exploratory behaviour spanning 30–120 s. A total of 10 min of active exploration was selected for each mouse.

EEG recordings from the cortex and hippocampus were first analyzed to detect time periods of strong theta oscillations, which were indicative of stable recordings during which the animal was exploring. To detect theta oscillations, we used the spectrogram method using discrete short-time Fourier transforms. More specifically, data sampled at 200 Hz were first low-pass filtered by a 100th order finite impulse response filter. Spectrograms were then built from the data set and separated into 1 s intervals, to which a Hamming window was applied. The discrete Fourier transforms were then evaluated over 2^14^ points with zero padding. Finally, for each time slot, the algorithm identified oscillations with the highest intensity in the theta frequency band (4–12 Hz) and that lasted at least 1 s. The power spectral density was normalized in amplitude from 0 to 1 and equalized with an exponential function with exponent 0.15 to improve its contrast to random noise. This analysis resulted in a list of theta oscillations for each extracted EEG recording. For each time period of theta oscillations, we then calculated the average power in the gamma frequency band. Analysis was performed in Matlab (7.11.0) (The Mathworks, Natick, MA, USA). Results are expressed as mean±s.e.m. The investigator was blind to the genotype until after data analysis was completed.

### Behavioural studies

All animals were kept under a light-dark cycle (12 h light–12 h dark) in a temperature and humidity controlled room. Food and water were available *ad libitum*. Room lights were kept low during all procedures. For all experiments, a camera was mounted above the arena; images were captured and transmitted to a computer running the Smart software (Panlab, Harvard Apparatus). The sequence of animals tested was randomized by the genotype. Care was taken to test litters containing both the genotypes specific to the breeding. Results are presented as mean ±s.e.m.

### Spontaneous open-field activity

The open-field test was performed as previously described^14^. Each subject (P25) was gently placed in the centre of the open-field, allowed to freely explore undisturbed for 10 min, after which the animal was removed, the arena cleaned with 70% ethanol and dried before testing the next animal. Locomotor activity was indexed as the total distance travelled (*m*).

### Elevated plus maze

Experiments were performed as described in ref. 67. The test session lasted 5 min during which the animals (P27) were allowed to explore the maze freely before being removed and returned to their home-cage. The percentage of time spent in the open arms (open/(open+closed) × 100) was scored as a measure of anxiety-related behaviour.

### Three-chamber

A three-chamber arena was used to assess the social recognition performance of the mice^68^. The tested animal (P36) was placed in the middle of the central chamber and allowed to explore all the chambers for 10 min. During this habituation session, small wire cages were present, one in each opposite chamber. After habituation, an unfamiliar conspecific of the same sex and age (Stranger 1) was placed inside a small wire cage whereas the other remained empty. The tested animal was allowed to freely explore the three chambers of the apparatus for 10 min. At the end of this 10 min, a new unfamiliar mouse of the same sex and the same age (Stranger 2) was placed in the previously unoccupied wire cage and the tested mouse was examined for an additional 10 min to assess preference for social novelty. Stanger 1 and stranger 2 animals originated from different home cages and had never been in physical contact with the tested mice or between each other. Social novelty preference was evaluated by quantifying the time spent by the tested mice in each chamber during the third 10 min session.

### T-maze

A discret-two-trials spontaneous alternation paradigm in a T-maze apparatus was used to assess the working memory^69^. Each test consisted of a two free-choice trials separated by a 1 min inter-trial interval (ITI) for 3 consecutive days. At the beginning of each trial, the animals (P38-40) were placed in the starting arm, allowed to freely move until one arm was chosen. The animals were left in the choice arm for 10 s before being removed and placed in the home-cage for 50 s. Then, the individuals were allowed to perform another free-choice trial. If the mice entered in the opposite arm than the first trial, the responses were considered as an alternation.

### Statistical analysis

Data were expressed as mean±s.e.m. unless otherwise specified in the legends. Normality tests were performed for all data analysed. Differences between two groups were assessed with the Student's *t*-test for normally distributed data or with the Mann–Whitney rank sum test for not-normally distributed data. Differences between multiple groups were assessed with one-way ANOVA with either Dunn's or Tukey's *post hoc* test for non-normally distributed and normally distributed data, respectively. Two-way ANOVA with Sidak's multiple comparison *post hoc* test was used for the detection of differences in the novelty social recognition test. Two-way Repeated Measure ANOVA with Bonferroni's multiple comparison *post hoc* test was used for analysing light-evoked IPSCs, considering N of mice as independent replicates. mIPSC frequency and amplitude were represented with Tukey boxplots (median±1.5 × interquartile range; box represents 1st–3rd quartile). In the same graph, a cross represented mean mIPSC frequency and amplitude values. In addition, all individual cell values were reported, using different symbols for different animals. mIPSC frequency and amplitude were analysed using a LMM, modelling animal as a random effect and genotype as fixed effect. We used this statistical analysis because we considered the number of mice as independent replicates and the number of cell in each mouse as repeated measures. For gamma oscillation power analysis, averages from the two genotypes were compared with Wilcoxon rank sum tests since values were not normally distributed. Finally, two-tailed power analysis was performed for all experimental data, using alpha=0.05 and desired power=0.8. Statistical analysis was performed using Prism 5.0 (GraphPad Software), SigmaStat 3.5 (Systat) and the function lmer implemented in the R package lme4 specifically for LMM.

Three animals were excluded from the analysis of electrophysiological recordings because the genotype was ambiguous (*n*=1 mouse) or tdTomato fluorescence was not detectable after AAV injection (*n*=2 mice).

### Data availability

Detailed statistics and data that support the findings of this study are available from the corresponding authors on request.

## Additional information

**How to cite this article:** Berryer, M. H. *et al*. Decrease of *SYNGAP1* in GABAergic cells impairs inhibitory synapse connectivity, synaptic inhibition and cognitive function. *Nat. Commun.* **7,** 13340 doi: 10.1038/ncomms13340 (2016).

**Publisher's note:** Springer Nature remains neutral with regard to jurisdictional claims in published maps and institutional affiliations.

## Supplementary Material

Supplementary Information Supplementary Figures 1-6
