# Supplementary material for: Decrease of SYNGAP1 in GABAergic cells impairs inhibitory synapse connectivity, synaptic inhibition and cognitive function
Source: Nat Commun. 2016 Nov 9;7:13340. doi: 10.1038/ncomms13340 (PMC5105197; doi:10.1038/ncomms13340)
Supplement: Supplementary Information — Supplementary Figures 1-6 [file ncomms13340-s1.pdf]

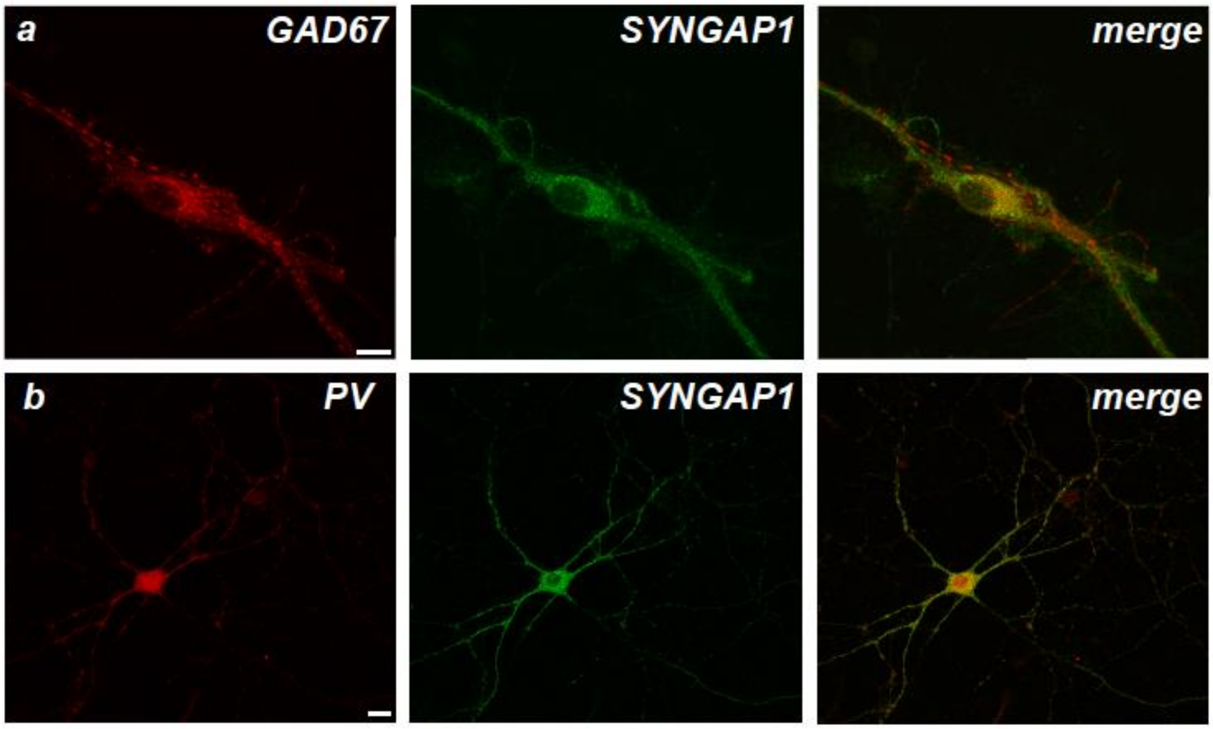

Supplementary Figure 1. GABAergic interneurons express Syngap1. (*a*, *b)* Dissociated cortical neurons from E18 rat embryos cultured for 21 days *in vitro* and immunostained for either (a) GAD67 (red) or (b) PV (red) and SYNGAP1 (green) show colocalization of both GAD67 and PV with Syngap1. Scale bar, 10µm.

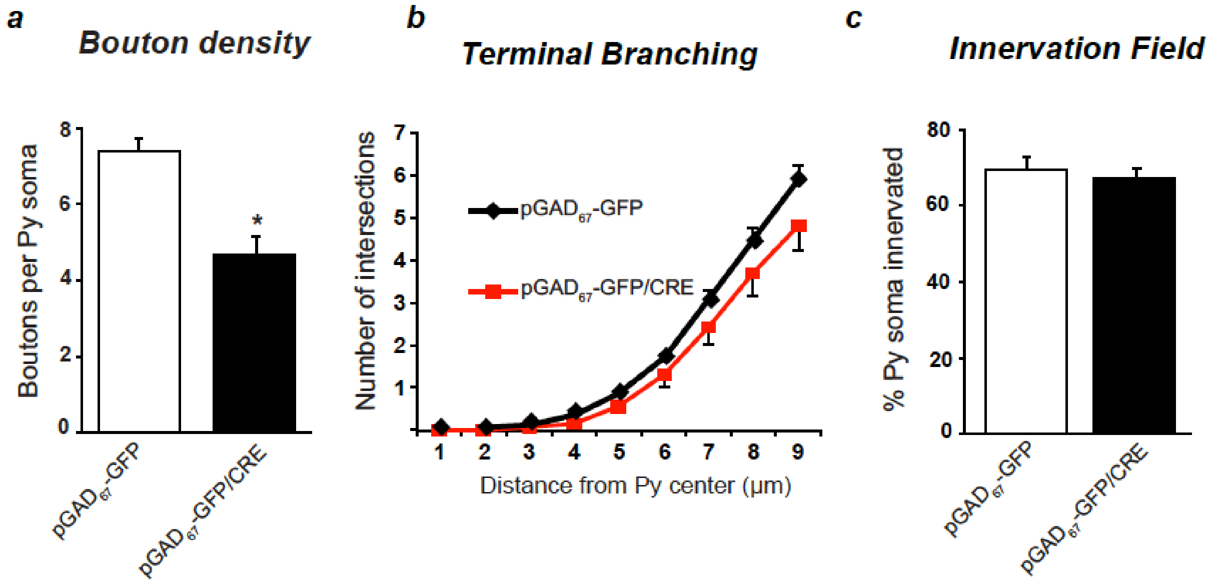

Supplementary Figure 2. Cre-mediated inactivation of *Syngap1* from EP16 to EP24 reduces basket cell bouton density. Cortical organotypic cultures prepared from *Syngap1*$^{flox/flox}$ mice were transfected at EP16 with $pGAD_{67}$-GFP or $pGAD_{67}$-Cre/GFP, fixed at EP24 and immunostained for NeuN. Perisomatic bouton density is significantly decreased in basket cells transfected with $pGAD_{67}$-Cre/GFP (*a;* Student's *t*-test *p=0.001), whereas the complexity of terminal axon branching (*b*, Student's *t*-test, p>0.1) (*b*) and the percentage of innervated cells (*c*, Student's *t*-test, p=0.286) are not affected compared to age matched control basket cells. n=8 basket cells trasfected with $pGAD_{67}$-GFP from n=4 mice and n=6 basket cells transfected with $pGAD_{67}$-Cre/GFP from n=4 mice.

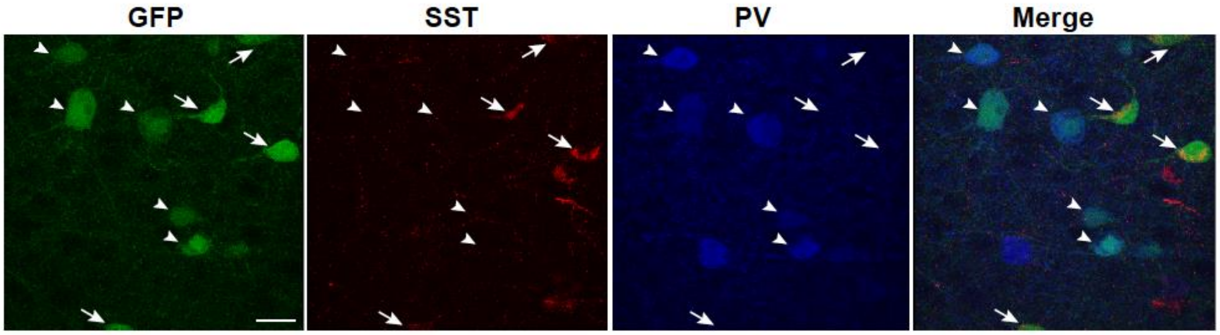

Supplementary Figure 3. Cre-mediated expression of GFP in Tg(*Nkx2.1-Cre*) ;*Syngap1*$^{flox/+}$*;RCE* mice is specific to SST+ and PV+ interneurons. Coronal section of somatosensory cortex (layer 5) from a Tg(*Nkx2.1-Cre*);*Syngap1*$^{flox/+}$*;RCE* mouse immunostained for PV (blue; mouse anti-PV, Swant 1:1000, #235) and SST (red; rabbit anti-SST, Santa-Cruz, 1:500; SC-13099). GFP+ cells express either PV (arrowheads) or SST (arrows), therefore confirming that Cre expression is specific to GABAergic interneurons derived from the MGE in this mouse model. Scale bar 20µm.

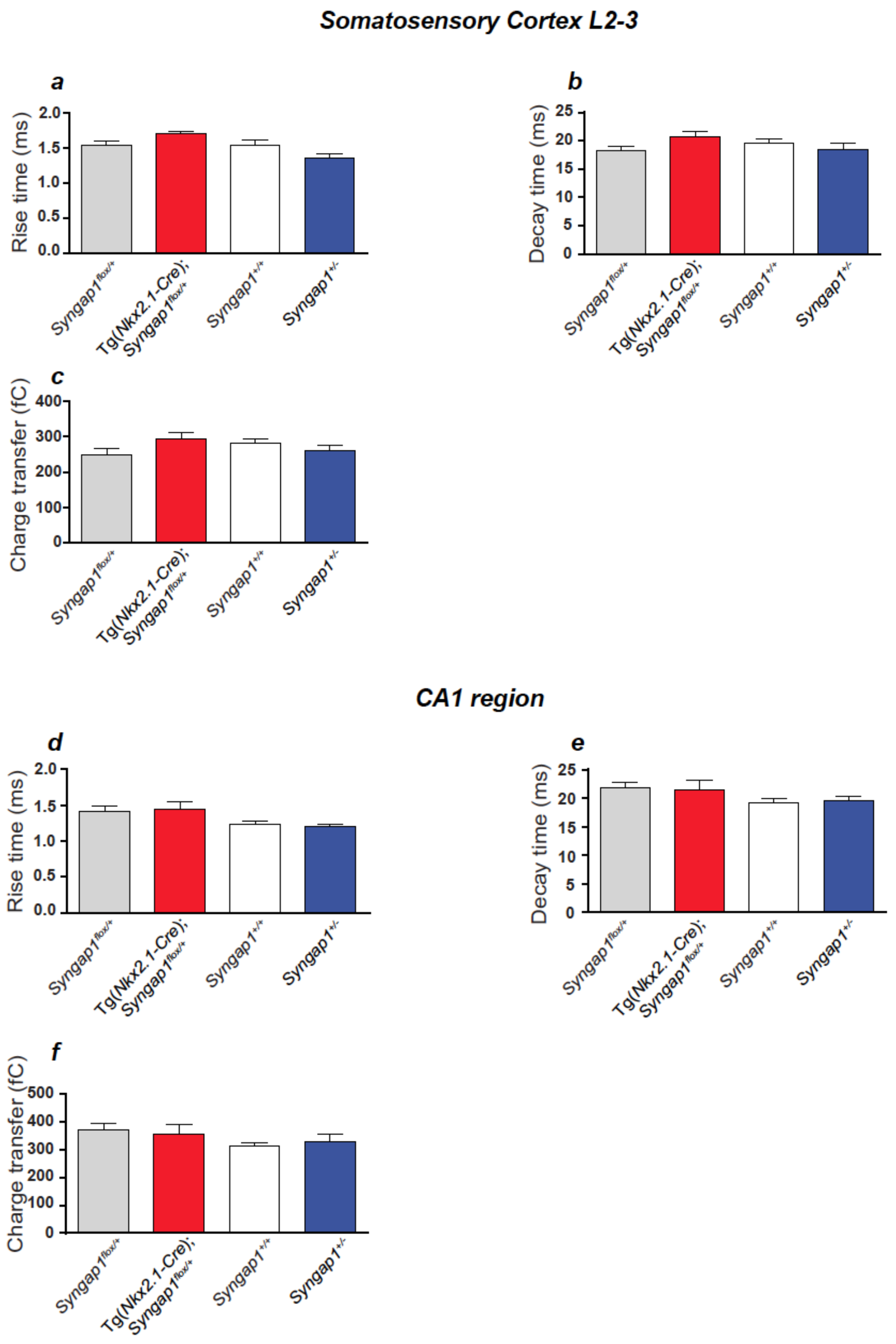

Supplementary Figure 4. *Syngap1* haploinsufficiency in MGE-derived interneurons does not affect mIPSC rise time, decay time and transfer charge. (*a*-c) Rise time, decay time and charge transfer of mIPSC recorded from L2-3 pyramidal neurons in adult somatosensory cortex. (*d*-f) Rise time, decay time and charge transfer of mIPSC recorded from CA1 pyramidal neurons. Somatosensory cortex L2-3: n=11 neurons from n=3 *Syngap1*$^{flox/+}$ mice; n=12 neurons from n=3 Tg(*Nkx2.1-Cre*);*Syngap1*$^{flox/+}$ mice; n=15 neurons from n=6 *Syngap1*$^{+/+}$ mice; n=14 neurons from

n=4 *Syngap1*$^{+/-}$. CA1: n=9 neurons from n=4 *Syngap1*$^{flox/+}$ mice; n=8 neurons from n=5 Tg(*Nkx2.1-Cre*);*Syngap1*$^{flox/+}$ mice; n=10 neurons from n=3 *Syngap1*$^{+/+}$ mice; n=9 neurons from n=3 *Syngap1*$^{+/-}$.

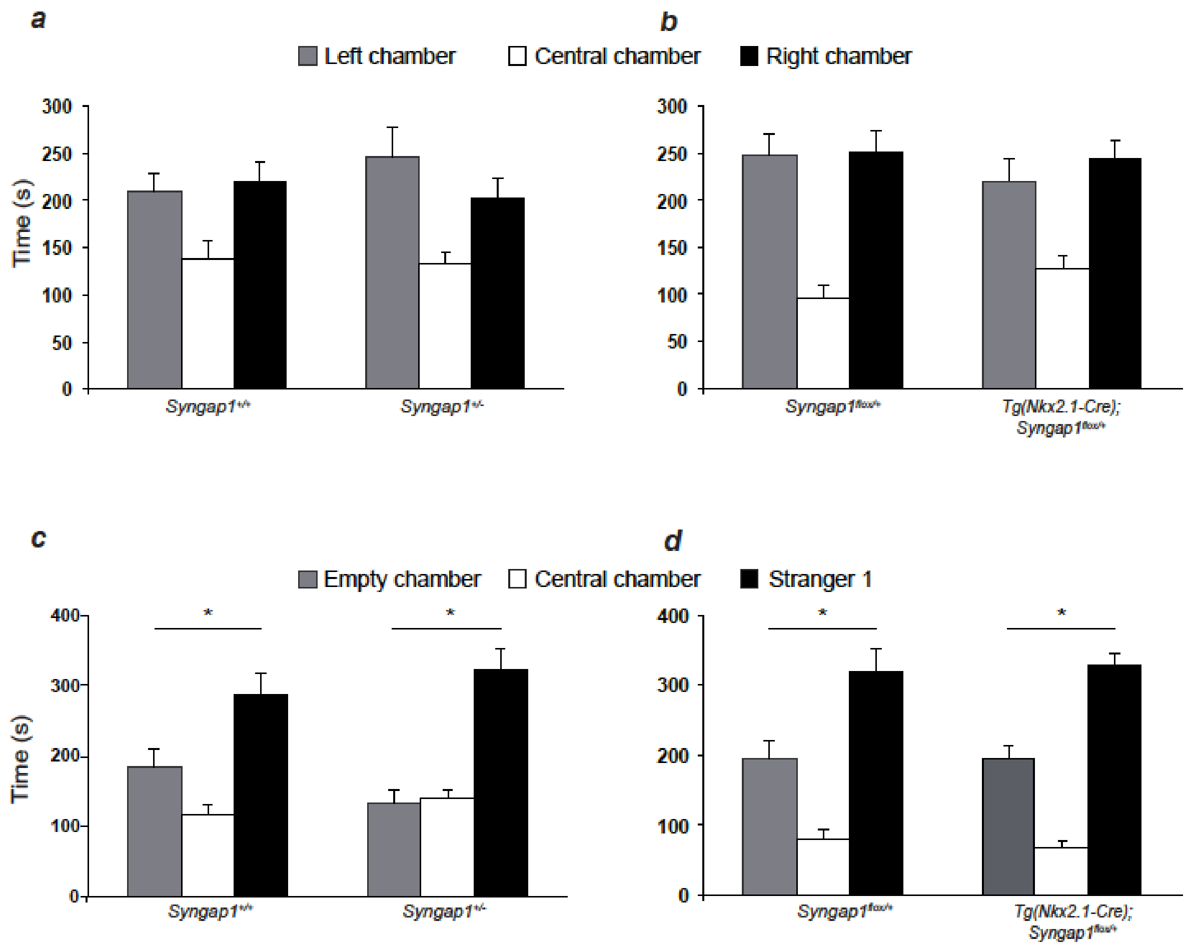

Supplementary Figure 5. *Syngap1*$^{+/-}$ and Tg(*Nkx2.1-Cre*);*Syngap1*$^{flox/+}$ mice show normal social approach behavior. (*a*) *Syngap1*$^{+/+}$, *Syngap1*$^{+/-}$, and (*b*) *Syngap1*$^{flox/+}$, Tg(*Nkx2.1-Cre);Syngap1*$^{flox/+}$ mice (n=10 for each genotype) were allowed to freely explore the social interaction apparatus for 10 min (habituation). Time (s) spent in the chambers was recorded. Neither genotype was demonstrated any preference for right or left chamber during habituation session (Right chamber X Left chamber X Genotype, Two-way ANOVA Sidak's multiple comparison *post-hoc* test, p>0.05). (*c*) *Syngap1*$^{+/+}$, *Syngap1*$^{+/-}$, and (*d*) *Syngap1*$^{flox/+}$ and Tg(*Nkx2.1-Cre);Syngap1*$^{flox/+}$ mice (n=10 for each genotype) were tested for sociability with an unknown conspecific (social interaction). Both genotypes showed a significant preference for the stranger 1 relative to the chamber containing the empty cage (Stranger 1 X Empty chamber X Genotype, Two-way ANOVA with Sidak's multiple comparison *post-hoc* test, *p<0.05).

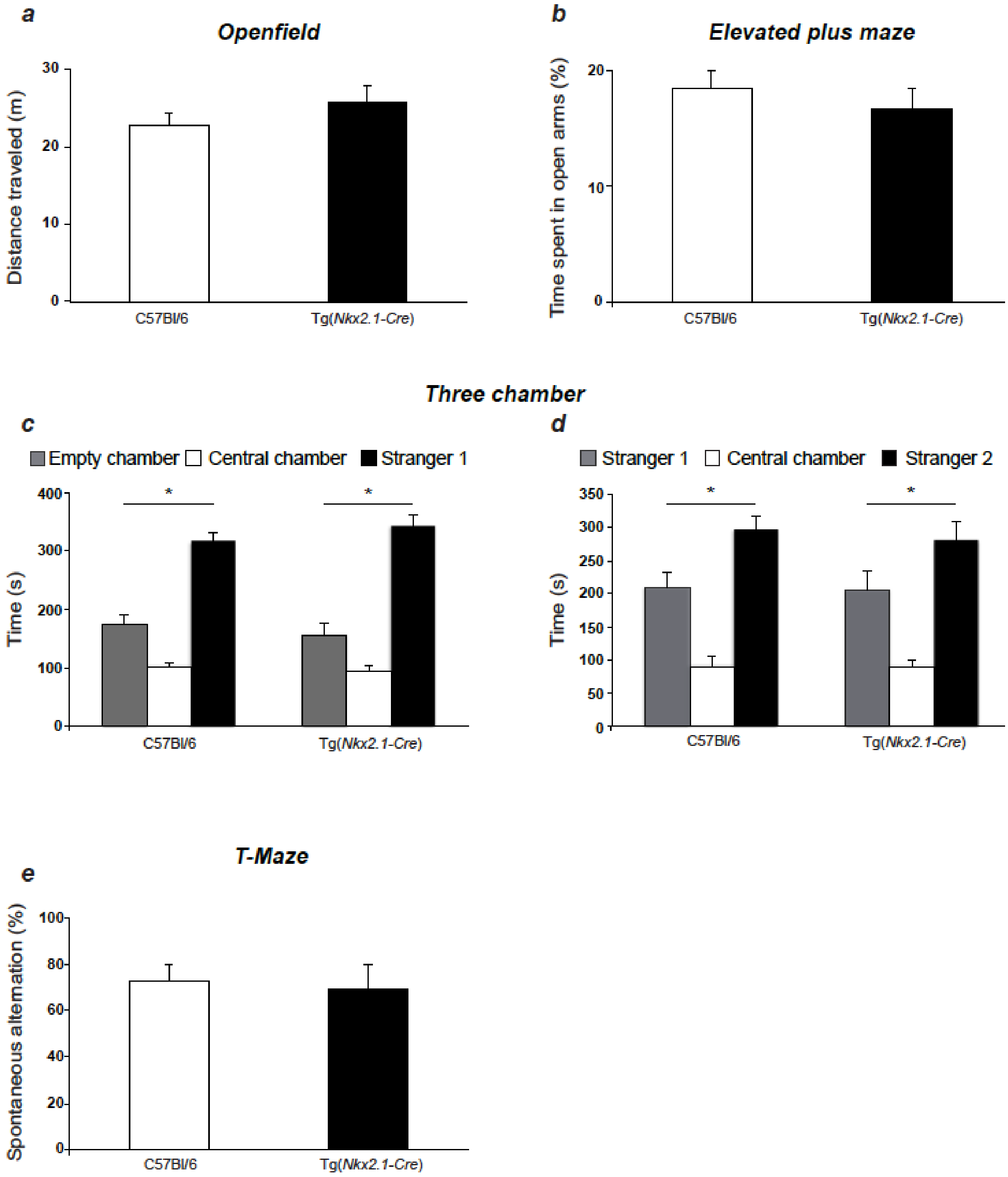

Supplementary Figure 6, *Tg(Nkx-Cre)* show normal locomotor, anxiety, spontaneous alternation and social behavior. (a, b) Comparison of Tg(*Nkx2.1-Cre*) mice with their control littermates (C57Bl6) show no statistical differences in open field (a) and elevated plus maze maze (b) (Student's *t*-test, $p>0.05$). n=6 C57Bl/6 mice; n=9 Tg(*Nkx2.1-Cre*) mice.

(c, d) In the 3-chamber test, Tg(*Nkx2.1-Cre*) mice show a significant preference for the stranger 1 relative to the chamber containing the empty cage (c) or for a novel mouse (Stranger 2) compared to a known mouse (stranger 1) (d), as is the case for wild-type mice (Stranger 1 X Empty chamber X Genotype, Two-way ANOVA with Sidak's multiple comparison post-hoc test, *$p<0.05$). n=12 C57Bl/6 mice; n=14 Tg(*Nkx2.1-Cre*) mice. (e) Tg(*Nkx2.1-Cre*) mice perform as well as their littermates on the T-maze test. (Student's *t*-test, $p<0.05$). n=10 C57Bl/6 mice; n=11 Tg(*Nkx2.1-Cre*) mice.
